# Silencing of A20 Aggravates Neuronal Death and Inflammation After Traumatic Brain Injury: A Potential Trigger of Necroptosis

**DOI:** 10.3389/fnmol.2019.00222

**Published:** 2019-09-19

**Authors:** Zhongyuan Bao, Liang Fan, Lin Zhao, Xiupeng Xu, Yinlong Liu, Honglu Chao, Ning Liu, Yongping You, Yan Liu, Xiaoming Wang, Jing Ji

**Affiliations:** ^1^Department of Neurosurgery, The First Affiliated Hospital of Nanjing Medical University, Nanjing, China; ^2^Department of Neurosurgery, The Third Affiliated Hospital of Nanchang University, Nanchang, China; ^3^Department of Neurosurgery, The Affiliated Suzhou Hospital of Nanjing Medical University, Suzhou Municipal Hospital, Suzhou, China; ^4^Institute for Stem Cell and Neural Regeneration, School of Pharmacy, Nanjing Medical University, Nanjing, China; ^5^Department of Immunology, Nanjing Medical University, Nanjing, China

**Keywords:** necroptosis, inflammation, traumatic brain injury, A20, neuronal death

## Abstract

Programmed cell death is an important biological process that plays an indispensable role in traumatic brain injury (TBI). Inhibition of necroptosis, a type of programmed cell death, is pivotal in neuroprotection and in preventing associated inflammatory responses. Our results showed that necroptosis occurred in human brain tissues after TBI. Necroptosis was also induced by controlled cortical impact (CCI) injury in a rat model of TBI and was accompanied by high translocation of high-mobility group box-1 (HMGB1) to the cytoplasm. HMGB1 was then passed through the impaired cell membrane to upregulate the receptor for advanced glycation end-products (RAGE), nuclear factor (NF)-κB, and inflammatory factors such as interleukin-6 (IL-6), interleukin-1 (IL-1β), as well as NACHT, LRR and PYD domains-containing protein 3 (NLRP3). Necroptosis was alleviated by necrostatin-1 and melatonin but not Z-VAD (a caspase inhibitor), which is consistent with the characteristic of caspase-independent signaling. This study also demonstrated that tumor necrosis factor, alpha-induced protein 3 (TNFAIP3, also known as A20) was indispensable for regulating and controlling necroptosis and inflammation after CCI. We found that a lack of A20 in a CCI model led to aggressive necroptosis and attenuated the anti-necroptotic effects of necrostatin-1 and melatonin.

## Introduction

Traumatic brain injury (TBI) causes high fatality and disability rates worldwide, especially among young people (McDonald et al., 2016). TBI is a complex condition initiated by mechanical tissue disruption, which is then followed by a secondary injury phase. During this phase, a variety of types of programmed cell death lead to neuronal loss (Dusick et al., 2012; Hinson et al., 2015). These events are sometimes accompanied by cognitive and neurological deficits in human TBI and in rat models of experimentally controlled cortical impact (CCI) injury (Li et al., 2013; Muccigrosso et al., 2016). During therapy, the pathway affected by the initial mechanical tissue disruption is refractory to treatment. Therefore, blockade of biochemical and cellular events of secondary injury—such as inflammation, autophagy, and apoptosis—is key to improving neurological outcomes (Wu et al., 2016; Cui et al., 2017).

Well-characterized types of programmed cell death include autophagy, apoptosis, pyroptosis and others (Elmore, 2007; Booth et al., 2014; Doitsh et al., 2014). In general, cell death can be categorized as either caspase-dependent or caspase-independent (Hirose and Horvitz, 2013). Recently, necroptosis has been suggested to be an important form of programmed cell death that is caspase-independent (Cabon et al., 2012). Necroptosis causes characteristic morphological changes such as defects in cell membrane integrity, mitochondrial swelling, and intact nuclear envelope (Sun et al., 2016). After necroptosis, inflammation occurs due to the release of inflammatory factors into the extracellular space from damaged and incomplete cell membranes. Clinically, necroptosis is activated by external stimuli or oxidative stress caused by stroke, ischemia/reperfusion injury, pancreatitis, cancer, or TBI (Koshinuma et al., 2014; Liu et al., 2016; Su et al., 2016; Wang et al., 2016; Zille et al., 2017). When TBI occurs, tumor necrosis factor (TNF) activates receptor-interacting protein 1 (RIP1), which is recruited to form a complex called the necrosome (Dondelinger et al., 2016). The formation of a necrosome is necessary for necroptosis and promote inflammation (Martens et al., 2017). TNF-induced protein 3 (TNFAIP3, also known as A20) is a protein that is implicated in many diseases and regulates cell fate *via* various pathways. Previous work has shown that A20-deficient mice develop massive inflammation, cachexia and die prematurely (Lee et al., 2000). It has also been shown that A20-deficient mice have markedly increased production of inflammatory cytokines and develop spontaneous arthritis, which is a finding that was shown to be associated with A20 limiting Toll-like receptor signaling in myeloid lineage cells (Matmati et al., 2011). A20 could inhibit TLR signal pathway not only in the spontaneous arthritis (Hammer et al., 2011), but in the lymphadenopathy, splenomegaly (Ma and Malynn, 2012) and neuroinflammation (Kinsella et al., 2018). In acute brain injury, upregulation of A20 alleviates inflammatory injury (Han et al., 2016; Zhan et al., 2016; Meng et al., 2017). In this rat CCI model, increased A20 was found. However, whether deficient A20 expression could exacerbate necroptosis and inflammatory responses following CCI have not been well-characterized.

A previous study reported that necrostatin-1 (Nec-1) prevented necroptosis by targeting RIP1 (Takahashi et al., 2012). Z-VAD, a broad-spectrum caspase inhibitor, blocks apoptosis rather than necroptosis. Z-VAD was also reported to prevent cell death of hippocampal neurons and white blood cell influx into the cerebrospinal fluid (CSF; Braun et al., 1999). Melatonin, an indoleamine that is mainly synthesized by the pineal gland, also exhibits neuroprotective effects (Calvo et al., 2013; Tian et al., 2014). Recent work has shown that melatonin inhibits necroptosis-associated inflammatory signaling and increases early growth response 1 and macrophage inflammatory protein 2 during liver fibrosis (Choi et al., 2015). It was also found that melatonin suppressed necroptosis by regulating A20 after intracerebral hemorrhage. However, it is unclear how melatonin regulates TBI-induced necroptosis.

In the present study, we first analyzed human brain samples to confirm that necroptosis occurs after TBI. Then, we used a CCI rat model to demonstrate that inhibition of both necroptosis and inflammatory responses by Nec-1 and melatonin contributed to neuroprotection that likely involved A20 (TNFAIP3).

## Materials and Methods

### Animals and Experimental Design

All animal experiments were approved by the Animal Management Rule of the Chinese Ministry of Health (documentation 55, 2001). Operational process in experiments was performed in accordance with the approved guidelines and the experimental protocol of Nanjing Medical University. There was no significant difference in body temperature, weight, feed intake of all rats before the experiment.

### Experimental Design 1

To explore the necroptosis at each time point after CCI, SD rats were randomly assigned into three groups: the control group (*n* = 5), the sham group (*n* = 5), and the CCI group (*n* = 30). The CCI group were divided into five subgroups (*n* = 6 for each time point) at 3, 6, 9, 12, 24, and 48 h after CCI, respectively. All rats were sacrificed at the planned time point and the cortical and hippocampus CA1 tissue samples were collected for subsequent analysis such as western blot and quantitative real-time PCR (qRT-PCR; Supplementary Figure S1A).

### Experimental Design 2

To investigate whether necroptosis inhibition at relative time point could reduce secondary injury after CCI, we used Nec-1, Z-VAD and Melatonin. SD rats were randomly assigned into five groups: sham group (*n* = 21), CCI group (*n* = 24), CCI+Nec-1 group (*n* = 24), CCI+Z-VAD group (*n* = 24), CCI+Melatonin group (*n* = 24). Then we detect the changes of cell death, necroptosis markers, tissue lesion and behavior *via* Western Blot, IF, IHC, HE, Brain water content, lesion volume analysis and behavior test (Supplementary Figure S1B). Tissue samples were collected at 6 h after CCI for Western Blot, IF, IHC, HE, Brain water content and lesion volume analysis.

### Experimental Design 3

To detect whether A20 affect CCI induced necroptosis and subsequent response, we used AAV-shA20 and AAV-shCtrl. SD rats were randomly assigned into four groups: sham group (*n* = 23), CCI group (*n* = 23), CCI+AAV-shCtrl (*n* = 23) and CCI+AAV-shA20 (*n* = 23). Tissue samples were collected at 6 h for Western Blot, IF, HE, Brain water content and lesion volume analysis except for behavioral test (Supplementary Figure S1C).

### Experimental Design 4

To test whether silencing A20 could counteract the anti-necroptotic role of Nec-1 and melatonin, SD rats were randomly assigned into six groups: sham+AAV-shCtrl group (*n* = 25), CCI+AAV-shCtrl group (*n* = 27), CCI+Nec-1+AAV-shCtrl (*n* = 27), CCI+Melatonin+AAV-shCtrl (*n* = 27), CCI+Nec-1+AAV-shA20 (*n* = 27), CCI+Melatonin+AAV-shA20 (*n* = 27). Tissue samples were collected at 6 h except for behavioral test (Supplementary Figure S1D). A schedule for AAV administration and drug treatment for experiment design 4 was displayed in Supplementary Figure S1E.

Melatonin (N-acetyl-5-methoxytryptamine, #M5250) was dissolved in 5% ethanol in saline (20 mg/kg) and injected into the left side of the peritoneum at 1 h before the CCI (Wong et al., 2014). Nec-1 (Selleck, Houston, TX, USA) and Z-VAD (BioVision, Mountain View, CA, USA) were dissolved in 10% dimethyl sulfoxide (Sigma-Aldrich). Rats was administered Nec-1 or Z-VAD *via* intracerebroventricular injection (coordinates: 0.8 mm posterior, 1.5 mm right lateral, and 4.0 mm ventral from Bregma). Infusion of 2 μg Z-VAD in 10 μl vehicle was conducted at a rate of 20 μl/h for 30 min (Li et al., 2014). Six microliter Nec-1 with concentration of 25 mM was used at a rate of 0.5 μl/min (Liu et al., 2016). In this study, experimenters made efforts to minimize the pain of rats.

### CCI Model

The CCI model was used to induce TBI as previously described (Lin et al., 2016; Xu et al., 2017). Eight-week-old male rats, purchased by The Model Animal Research Center of Nanjing University (Nanjing, China), were anesthetized with 4% isoflurane in 70% N_2_O and 30% O_2_. Anesthesia was maintained using 2% isoflurane. The rats were then positioned in a stereotaxic frame. After the scalp and fascia were retracted, an 8-mm-diameter hole was drilled on the right cerebral hemisphere to expose the intact dura. For TBI administration, rats were subjected to CCI with a 6-mm metal impounder at 6.0 ± 0.2 m/s speed, 2.5 mm depth, and 50 ms hold duration. After the CCI, the scalp incision was sutured. Rats in the sham group were subjected to all of these procedures but were not subjected to CCI.

### Human Brain Tissues and Study Approval

Thirteen human brain tissues were obtained from The First Affiliated Hospital of Nanjing Medical University. Four normal brain tissues (NBTs) were obtained from patients who underwent surgery for cerebrovascular malformation, through which peripheral brain tissues were isolated. Nine TBI tissues were from patients with brain injury caused by accidents. The Research Ethics Committee of Nanjing Medical University approved the use of the human brain tissues, and the procedures were performed following the approved guidelines. Express permission from participants was obtained, and patients provided informed consent.

### AAV-shA20 Intracerebroventricular Injection

An adeno-associated shA20 virus targeting A20 was chemically synthesized by Genechem (Shanghai, China). Virus serotype is 9. The vector used was AAV9- U6-MCS-CAG-puro and U6 is promoter. The target sequences were as follows: No. 1, 5′-GCCCTGAAAT TCGAGCTGTTC-3′; No. 2, 5′-GCACGACTCA CCTGATCAATG-3′; and No. 3,5′-GCAGTGAGGA ACTCTGTATGG-3′. The AAV-shCtrl was generated after cloning short-hairpin RNA (shRNA) fragments into the adeno-associated virus (AAV) vector GV478 (Shanghai Genechem Company Limited). AAV packaging was performed by cotransfecting AAV-293 cells with the recombinant AAV vector, pAAV-RC vector, and pHelper vector. AAV were collected from the AAV-293 cell supernatant, condensed, and purified for further animal experiments. Virus titer is 10^12^ V.G./ml. Intracerebroventricular injection of AAV-shA20 was performed as previously described (Stengel et al., 2011). Rats were anesthetized persistently by 2% isoflurane. They were placed in a stereotaxic apparatus. Injection coordinates were 0.8 mm posterior, 1.5 mm right lateral, and 4.0 mm ventral from Bregma (Manolidis et al., 2004). A chronic guide cannula (Plastics One Inc., Roanoke, VA, USA) was implanted with into the selected point and secured by dental cement. Five microliter AAV was injected in each rat brain. After intracerebroventricular injection, anesthetized rats were revived within 5 min. Ten days were needed for successful transfection before CCI treatment.

### Immunoblotting

Only the ipsilateral hemisphere tissues including cortex and hippocampus CA1 were collected. Brain tissues from each rat were obtained timely on ice. Protein was extracted using RIPA buffer (Sigma-Aldrich). A subcellular fraction protein extraction kit (KGP350-1, Keygen, Nanjing, China) was used for subcellular fractionation and the membrane and cytoplasmic extracts were subjected to immunoblotting. The protein concentration was determined with a bicinchoninic acid protein assay kit (Thermo Fisher Scientific, Waltham, MA, USA). The proteins were separated by 10%–15% SDS–PAGE gels or Phos-tag™ SDS–PAGE and transferred onto polyvinylidene fluoride (PVDF) membranes. The PVDF membranes were blocked in 5% nonfat dried milk for 2 h at room temperature (RT) and subsequently probed with specific antibodies overnight at 4°C. ImageJ was used to analyzed the protein band densities. The antibodies used were specific for the following proteins: β-actin (1:2,000, Beyotime, AF0003), interleukin-1 (IL-1β; 1:1,000, Abcam, ab9722), cleaved caspase-3 (1:1,000, Cell Signaling Technology), GAPDH (1:2,000, Beyotime, AF0006), H3 (1:2,000, Cell Signaling Technology, #4499), Interleukin-6 (IL-6; 1:1,000, Novus, NB600-1131), NLRP3 (1:1,000, Novus, NBP2-12446), RIP1 (1:1,000, Novus, NBP1-77077), RIP3 (1:1,000, Abcam, ab62344), mixed lineage kinase domain-like protein (MLKL; 1:1,000, Abcam, ab184718), MLKL (1:500, Santa Cruz Biotechnology, sc-165024), high-mobility group box-1 (HMGB1; 1:1,000, Abcam, ab79823), receptor for advanced glycation end-products (RAGE; 1:1,000, Abcam, ab3611), p65 (1:1,000, Cell Signaling Technology, #9936) and TNFAIP3 (1:1,000, Novus, NBP1-77024).

### Quantitative Real-Time PCR

Total RNA was extracted from the brain tissue of rats using RNAiso Plus (Total RNA Extraction Reagent; Takara Bio Inc., Tokyo, Japan) in accordance with the manufacturer’s protocol. The RNA was reverse transcribed by oligodeoxythymidine primer using a PrimeScript RT Reagent Kit. Primer list and efficiencies were provided in Table 3.

**Table 1:** Information regarding nontraumatic samples.

No.	Age	Gender	Area
1	53	Male	Right temporal lobe
2	47	Female	Left inferior prefrontal
3	44	Female	Left temporal lobe
4	38	Female	Left inferior prefrontal

*Four nontraumatic brain tissues from cerebrovascular-malformation patients were described according to the age, gender and area*.

**Table 2:** Information regarding TBI samples.

TBI sample number	Accident	Injury area	Gender	Age	GCS scores
1	Traffic accident	Left temporal lobe	Male	42	9
2	Falling injury	Occipital lobe	Female	70	8
3	Impact damage in soccer game	Left parietal lobe	Male	27	7
4	Traffic accident	Right temporal lobe	Male	52	10
5	Traffic accident	Left temporal lobe and occipital lobe	Female	58	5
6	Fight accident	Right parietal lobe	Male	33	8
7	Traffic accident	Left parietal lobe and contralateral encephalocele	Female	60	7
8	Traffic accident	Left temporal lobe	Male	47	8
9	Traffic accident	Left temporal lobe	Female	55	8

*Nine TBI samples were described according to the kind of accident, injury area, as well as the patient gender, age and GCS scores*.

**Table 3:** Information regarding primers.

Gene	Primer	Efficiency (E)
RIP1	F: 5′-AGGTACAGGAGTTTGGTATGGGC-3′ R:
	5′-GGTGGTGCCA AGGAGATGTATG-3	92.33%
RIP3	F: 5′-CTGTCGCCTGCTAGAGGAAG-3′ R:
	5′-TCTGCTAACTTGGCGTGGAG-3′	94.16%
MLKL	F: 5′-CCCGAGTTGTTGCAGGAGAT-3′ R:
	5′-TCTCCAAGATTCCATCCGCAG-3′	93.67%
β-actin	F: 5′ -AGGGAAATCGTGCGTGACAT-3′ R:
	5′-AGGGAAATCGTGCGTGACAT-3′	95.33%

Primer list and efficiencies were provided. Formula E = [10^∧(−1/slope)^ − 1]*100% was used to measure the efficiency.

### Co-immunoprecipitation

Total cell lysates from traumatic brain tissues were harvested using weak RIPA lysis buffer (Cell Signaling Technology, Danvers, MA, USA), and were pre-cleared with 50% protein A/G agarose for 1 h. Then 500 μl of extracted proteins were incubated with 2 μg primary antibody overnight at 4°C. The immune complexes were pulled down with protein A/G agarose for 4 h in a 4°C shaker. Microbeads were collected and washed, and then proteins were eluted through boiling in 1× loading buffer followed by immunoblotting analysis.

### Cerebrospinal Fluid (CSF) Extraction and Enzyme-Linked Immunosorbent Assay (Elisa)

Six hours after CCI, the CSF of rats was collected. In brief, the rats were anesthetized and mounted in a stereotaxic frame. The skin over the cisterna magna was shaved and disinfected with povidone–iodine. A midline sagittal incision was made over the dorsal aspect of the hindbrain, and three layers of muscle were carefully peeled back to expose the cisterna magna. About 60 μL of CSF was extracted slowly using a syringe needle. The HMGB1 protein level in the CSF was tested using commercial ELISA kits (ab18256). Sample analysis was conducted according to the instruction provided. Samples were diluted using a 1× diluent solution. Dilution ratio in the rat sample was 1:20.

### Immunofluorescence and Terminal Deoxynucleotidyl Transferase dUTP Nick-End Labeling Staining

Briefly, frozen brain tissues were sliced into 8-μm-thick sections. After that, sections were rinsed in 4% paraformaldehyde for 30 min at RT. Then, the sections were incubated with 5% goat serum and 0.2% Triton X-100 diluted in PBS for 1 h at RT. Next, the sections were incubated with anti-HMGB1 (ab18256, Abcam) in dilution buffer overnight at 4°C. After being rinsed in PBS for three times, the sections were incubated for 1 h at RT with secondary antibodies conjugated to Alexa Fluor^®^ 488 or Cy3. Nuclei were stained with DAPI. DNA fragmentation experiment, indicative of cell death, was detected using terminal deoxynucleotidyl transferase dUTP nick-end labeling (TUNEL) assay. Double labeling with cleaved caspase-3 (red) and TUNEL (green) staining was applied to a series of sections according to the manufacturer’s protocol (#12156792910; Roche, Mannheim, Germany). The same protocol was used to double-label iba-1 (green) and NLRP3 (red), iba-1 (green) and IL-1β (red), and GFAP (green) and IL-6 (red). The slides were imaged with an inverted fluorescence microscope (Leica DM 5000B; Leica, Wetzlar, Germany). Immunofluorescence was quantified using ImageJ and the background was subtracted. Each section was imaged randomly by scanning 5–8 fields in each quadrant.

### Immunohistochemistry and Hematoxylin and Eosin Staining

Briefly, fresh rat brain specimens obtained were processed into 8-μm-thick frozen sections. Immunohistochemical staining with a streptavidin-biotin immunoperoxidase assay was performed using antibodies against RIP1, RIP3 MLKL, and A20 (TNFAIP3). Hematoxylin and eosin (H&E) staining was carried out in the prepared 8-μm-thick sections. Slides were imaged under a light microscope (Leica).

### Brain Water Content and Cortical Lesion Volume Analysis

After euthanasia, rat brains were immediately obtained and weighed. Next, they were dried at 70°C in drying box for 3 days to obtain the dry weights. Water content (%) = [(wet weight − dry weight)/wet weight] × 100%. Lesion volumes were assessed as previously reported (Lin et al., 2017). One section was obtained every 0.5 mm for lesion volume measurement. Areas of defect on each slice were measured with ImageJ. Multiplying defected areas by 0.5 mm got the lesion volume of each slice. The total lesion volume was calculated from the summation of the lesion volume of each slice. From the anterior to the posterior limits of the lesion, an average of nine slices were collected and analyzed.

### Inverted Screen Test

In the inverted screen test, rats were placed on a wire grid (2 × 2 cm). Their forepaws and back claws held onto the wire, which was then inverted over a foam pad. The latency to fall (seconds) was recorded.

### Balance Beam Test

6-point scale method and four consecutive trials per session were used to evaluate for the rat motor ability (Esenaliev et al., 2018). The rats were trained to balance for 60 s on a short wooden beam (50 × 1.5 × 4 cm) raised 90 cm off the floor. According to their behavioral performance, the scale was recorded. The criteria are as follows:

1 = Balances with steady posture (grooms, climbs barrier).
2 = Balances with unsteady posture (grasps sides of beam and/or has shaky movements).
3 = Hugs the beam or slips or spins on the beam.
4 = Attempts to balance, but falls off after 10 s.
5 = Drapes over or hangs from the beam, falls off in less than 10 s.
6 = Falls off, making no attempt to balance or hang onto the beam.

### Morris Water Maze (MWM) Test

The same person performed relative parts of this experiment at the similar time each day. The pond was divided into four positions. Water temperature was maintained at 24°C. Start position sets were semi-random, which means unaltered sets from each of the four positions each day. Rats were allowed a maximum of 100 s to find the submerged platform. If the rats could not reach the platform within 100 s, they were forced to be placed on the platform for 15 s (Lin et al., 2017). The latencies to reach the platform were recorded and analyzed by software (CHROMOTRACK 3.0; San Diego Instruments, San Diego, CA, USA). When rats performed the Morris water maze (MWM) test, they meet the requirements to obtain brain samples and were sacrificed. Pentobarbital sodium (150–200 mg/kg) intraperitoneal injection was used to euthanize rats.

### Extraction of Primary Neuron and Astrocyte and Cell Culture

Newborn mice were disinfected with 75% alcohol and their heads were quickly severed. Their skull and perichondrium were dissected in a 4°C pre-cooled buffer under a dissecting microscope. Buffer formulation is 10% horse serum, 2% penicillin and streptomycin in 1:1 F12-DMEM. The brain tissue was then cut up and digested with 0.25% trypsin for 30 min. Buffer of triple volume stopped digestion. After the residue was filtered through a strainer, the remaining liquid was centrifuged at 1,000 rpm for 5 min. Remove the supernatant, re-suspend the precipitate with buffer, and lay the board which was coated by poly-L-lysine. The 6-well plate must have 7× 100,000 cells per well. After 5 h, buffer was instead by the neurobasal medium containing 2% B27 and 0.5 mmol/l glutamate at 37°C and 5% CO_2_. As for the astrocyte, cell suspension after filtration was plated onto poly-L-lysine-coated 75-cm^2^ flasks at 37°C and 5% CO_2_. The culture medium is 1:1 DMEM/F-12 with 10% fetal bovine serum (FBS, Invitrogen, Carlsbad, CA, USA) and 1% antibiotic–antimycotic. After the first day, astrocyte stick to the bottom of the bottle. Then, the cell cultures were shaken overnight at 37°C and 280 rpm on a tabletop shaker (Infors HT, Bottmingen, Switzerland) in order to minimize oligodendrocyte and microglia contamination. Discard the medium containing floating oligodendrocyte and microglia and obtain the primary astrocyte. HAPI, a rat microglia cell line, was obtained from ATCC (CRL-2815). It is cultured in DMEM+10% FBS at 37°C and 5% CO_2_.

### Cell Transfection

Small interfering RNAs (siRNAs) targeting A20 were purchased from GenePharma (Shanghai, China). The target sequences were as follows: 5′-GCAGTGAGGA ACTCTGTATGG-3′. Eighty percent cell density is suitable to perform transfection with si-A20 and si-NC. All oligonucleotides and plasmids were transfected into cells using Lipofectamine 2000 transfection reagent (Invitrogen, Carlsbad, CA, USA) following the manufacturer’s protocol. Transfection efficacy was assessed by western blotting.

### CCK-8 Assay

The cellular viability of primary neuron was estimated using CCK-8 (Beyotime, China). The primary neuronal cells in every group were seeded into 96-well cell culture plates. Every well contained 4 × 10^3^ cells in 100 μl culture media. At 0 h, 12 h and 24 h after TZ administration, the medium of each well was replaced with 100 μl fresh medium with 10% CCK8, and then the cells were incubated at 37°C for an additional 1 h. The absorbance was measured at 450 nm wavelength.

### Statistical Analysis

Data were analyzed with GraphPad 6.0 Software and reported as the mean ± standard error of the mean. An unpaired *t*-test or one-way analysis of variance (ANOVA) plus Tukey’s test was applied in western blot, fluorescence, brain water content, inverted screen test, and cortical lesion volume experiments to compare the differences between two groups. For behavioral tests, including the balance beam and MWM assays, data of the whole group were analyzed using two-way ANOVA for between-group comparisons. One-way ANOVA followed by Tukey *post hoc* test was used to analyzed the difference of groups at the same time point (* or # was labeled upon the relative time points). Significant differences were set at *P* < 0.05.

## Results

### Increased RIP1, RIP3 and MLKL Are Detected in Human TBI Tissues

**Figure 1:**
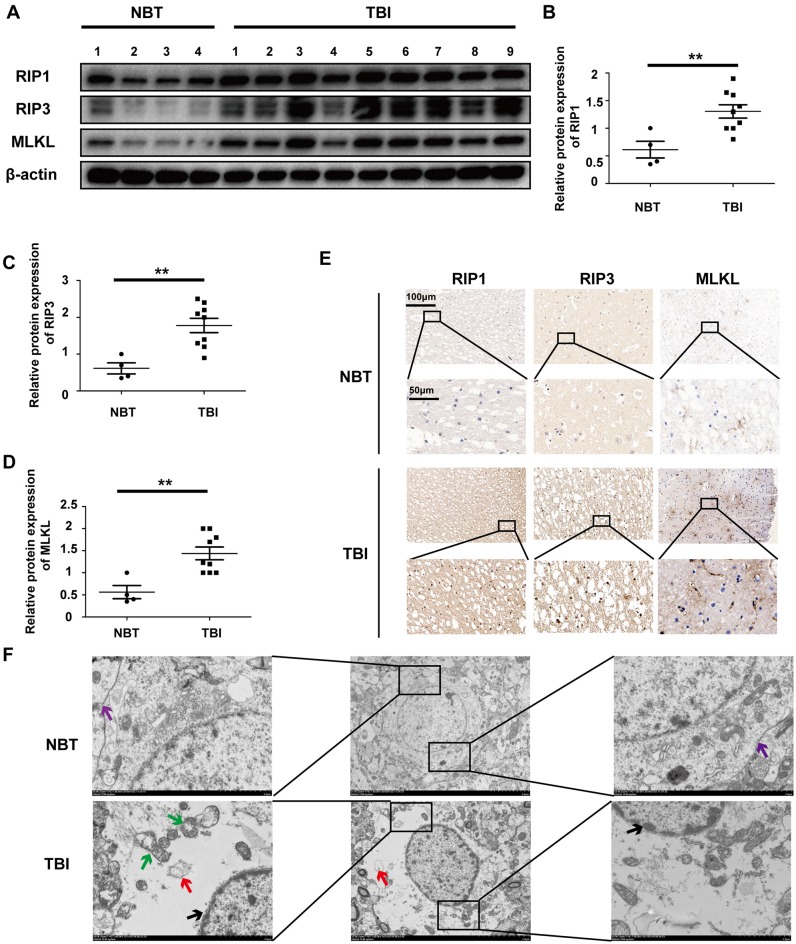
Traumatic brain injury (TBI) tissues show increased necroptosis compared with normal brain tissues (NBTs). **(A)** The protein expressions of receptor-interacting protein 1 (RIP1), RIP3 and mixed lineage kinase domain-like protein (MLKL) were analyzed in human NBT (*n* = 4) and TBI tissues (*n* = 9) *via* western blotting. β-actin was used as a control. **(B–D)** Protein expression of RIP1, RIP3 and MLKL was analyzed by statistical. **(E)** The expressions of RIP1, RIP3 and MLKL were tested in NBT and TBI tissues from Jiangsu Province Hospital by immunohistochemistry. **(F)** Electron microscopy was used to examine human normal brain and TBI tissues. Intact cell membrane (violet arrow) is labeled in NBT. Complete and continuous nuclear membrane (black arrow), swollen mitochondria (green arrow) and vacuoles (red arrow) are labeled in TBI tissues. All data were analyzed by one way analysis of variance (ANOVA) plus Tukey’s test. **P < 0.01 vs. NBT group.

Necroptosis has been observed in animal models of stroke, ischemia/reperfusion injury, and fracture. However, this process has not been investigated in human tissues, particularly for TBI. Therefore, we collected 13 human brain tissues, including four NBTs from cerebrovascular-malformation patients and nine TBI tissues from patients with brain injury caused during accidents. The surrounding NBTs were isolated and obtained during surgery performed to correct cerebrovascular malformations. A detailed description of the human brain tissues including the nontraumatic (NBTs) and TBI is shown in Tables s 1, 2. To assess the level of necroptosis in these tissues, we performed Western blotting for RIP1, RIP3, and MLKL. The levels of all three necroptotic markers were increased in TBI tissues compared to those in NBTs (Figure 1A); the analysis is shown in Figures 1B–D. Immunohistochemistry confirmed these results (Figure 1E). Electron microscopy indicated that, while the NBTs still had intact cell membranes, TBI tissues displayed morphological features of necroptosis, including mitochondrial swelling, fragmentary cell membranes, complete and continuous nuclear membranes, and vacuolization (Figure 1F). Therefore, these findings suggest that TBI triggers necroptosis.

### Protein Levels of Necrosome Components Are Elevated in a CCI Rat Model of TBI

**Figure 2:**
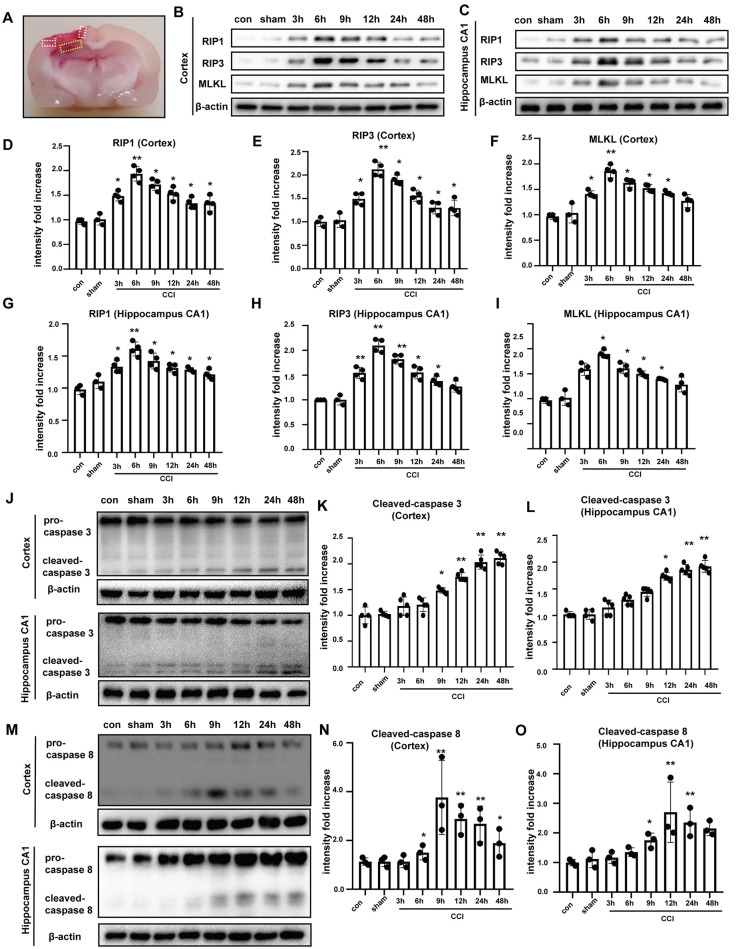
Tissues were obtained to perform western blot assays. **(A)** Location of collected tissues was labeled. Collected cortical tissues and hippocampus CA1 were marked by white and yellow frame, respectively. RIP1, RIP3 and MLKL in the **(B)** cortex and **(C)** hippocampus CA1 were examined *via* western blot from 0 h to 48 h after controlled cortical impact (CCI). Protein expression of RIP1, RIP3 and MLKL in the **(D–F)** cortex and **(G–I)** hippocampus CA1 from 0 h to 48 h after CCI was analyzed by statistical. Values are represented as means ± SEM (*n* = 3–4). **(J)** Cleaved caspase-3 was detected in cortex and hippocampus CA1 *via* western blotting from 0 h to 48 h after CCI. **(K,L)** Protein expression of cleaved caspase-3 in the cortex and hippocampus CA1 from 0 h to 48 h after CCI was measured. Values are represented as means ± SEM (*n* = 4–5). **(M)** Cleaved caspase-8 was detected in cortex and hippocampus CA1 *via* western blotting from 0 h to 48 h after CCI. **(N,O)** Protein expression of cleaved caspase-8 in the cortex and hippocampus CA1 from 0 h to 48 h after CCI was analyzed. Values are represented as means ± SEM (*n* = 4–5). β-actin was used as a control in western blot assays. All data were analyzed by one way ANOVA plus Tukey’s test. *P < 0.05 and **P < 0.01 vs. sham group.

To further investigate TBI-induced necroptosis in an experimental model, we examined brain tissues from rats subjected to CCI at different times post-CCI (3, 6, 9, 12, 24 h and 48 h) *via* Western blotting. The location of collected brain tissues was shown (Figure 2A). Compared to corresponding protein levels in controls, RIP1, RIP3, and MLKL were increased in cortical tissues since 3 h post-CCI and peaked at 6 h after CCI (Figures 2B,D–F). Similar results were observed in tissues from the hippocampal CA1 region (Figures 2C,G–I), which is closely linked to spatial learning and memory. mRNA levels of RIP1, RIP3 and MLKL were tested by qRT-PCR assay, showing that only the expressions of the three at 6 h were increased in cortex (Supplementary Figures S2A–C) and hippocampus CA1 (Supplementary Figures S2D,E). Necroptosis is a caspase-independent process (Delavallée et al., 2011). Therefore, we measured the protein levels of cleaved caspase-3 and cleaved caspase-8 in the cortex and in hippocampal tissues. Cleaved caspase-3 was not significantly increased at 6 h after CCI, but increased gradually and peaked at 24 h and 48 h after CCI in the cortex and hippocampal CA1, respectively (Figures 2J–L). Cleaved caspase-8 upregulation was found starting at 6 h after CCI, peaked at 9 h, and then decreased gradually in the cortex. In the hippocampus CA1, cleaved caspase-8 was increased at 9 h and peaked at 12 h after CCI (Figures 2M–O). These results indicated that caspase-3 and caspase-8 activation were not synchronized to the earlier increases in RIP1, RIP3, and MLKL. Additionally, 6 h after CCI, the phosphorylation levels of RIP3 and MLKL were significantly increased (Supplementary Figures S3A–C). Combination of RIP1/RIP3 and MLKL was tested by co-immunoprecipitation assays (Supplementary Figure S3D). At 6 h after CCI, RIP1, RIP3 and MLKL were increased (Supplementary Figure S3E) and RIP3 combined with more RIP1 and MLKL [Supplementary Figures S3D (middle) and S3F]. At the same time, MLKL could combine with more RIP1 and RIP3 [Supplementary Figures S3D (right) and S3G]. The abundant combination of RIP1/RIP3 and MLKL indicated that necroptosis was activated at 6 h after CCI. Thus, it is likely that CCI-induced necroptosis is independent of apoptosis. Additionally, we examined the expression of inflammatory factors. NLRP3, IL-6, and IL-1β were induced in the cortex (Supplementary Figures S3H–K) and CA1 (Supplementary Figures S3L–O). Furthermore, after CCI procedure, fragmentary cell membranes, intact nuclear membranes, and swollen mitochondria and vacuoles were observed by electron microscopy (Supplementary Figure S3P). These results verify that our CCI rat model leads to necroptosis and neuroinflammation.

### Nec-1 and Melatonin Prevent Upregulation of Necroptosis Effectors RIP1, RIP3 and MLKL Following CCI

**Figure 3:**
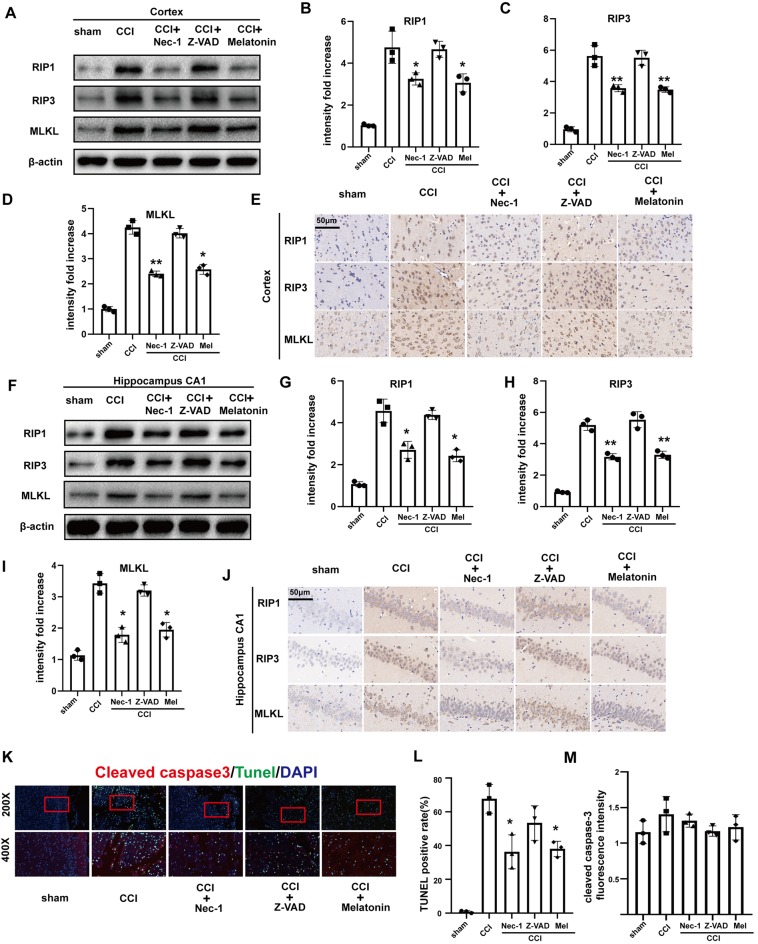
Effect of Nec-1, Z-VAD and melatonin on necroptosis. **(A)** At 6 h after CCI, RIP1, RIP3 and MLKL protein levels in the cortex detected by western blotting were decreased in Nec-1 and melatonin pretreatment groups, but there was no change in the Z-VAD pretreatment group. **(B–D)** Protein expression of RIP1, RIP3 and MLKL was analyzed by statistical. **(E)** Immunohistochemistry assays examined the effect of Nec-1, Z-VAD and melatonin on cortex RIP1, RIP3 and MLKL, respectively. **(F)** At 6 h after CCI, RIP1, RIP3 and MLKL protein levels in the hippocampus CA1 detected by western blotting were decreased in the Nec-1 and melatonin pretreatment groups, but not the Z-VAD pretreatment group. **(G–I)** Protein expression of RIP1, RIP3 and MLKL was analyzed by statistical. **(J)** Immunohistochemistry assays examined the effect of Nec-1, Z-VAD and melatonin on RIP1, RIP3 and MLKL in hippocampus CA1, respectively. **(K)** TdT-mediated dUTP Nick-End Labeling (TUNEL; green) and cleaved caspase-3 (red) dual immunofluorescent labeling was used and were analyzed by statistical **(L,M)** in the five groups. Values are represented as means ± SEM (*n* = 3). β-actin was used as a control in western blot assays. All data were analyzed by one way ANOVA plus Tukey’s test. *P < 0.05 and **P < 0.01 vs. CCI group.

Nec-1, melatonin, and Z-VAD have all been demonstrated to exert neuroprotective effects in previous studies (Li et al., 2014; Wang et al., 2014; Chetsawang et al., 2017). Here, we compared the effects of Nec-1 (an inhibitor of necroptosis), melatonin, and Z-VAD (an irreversible apoptotic inhibitor) on CCI-induced necroptosis. Western blotting (Figures 3A–D,F–I) and immunohistochemistry (Figures 3E,J) demonstrated that CCI-induced increases in RIP1, RIP3, and MLKL were attenuated in the cortex and CA1 *via* Nec-1 treatment. Melatonin produced similar results, indicating that melatonin may also inhibit the necroptotic process. However, there was no clear attenuation of the RIP1/RIP3/MLKL pathway after treatment with Z-VAD, confirming that activation of the RIP1/RIP3/MLKL pathway is caspase-independent.

To further investigate the effects of Nec-1, melatonin, and Z-VAD on cell death in the ipsilateral cortex and CA1 at 6 h after CCI, we performed TUNEL and cleaved caspase-3 dual-immunofluorescent labeling (Figures 3K–M). Cells that were positive for both TUNEL and cleaved caspase 3 were considered to be apoptotic cells. In contrast, cells that were positive for TUNEL but negative for cleaved caspase-3 were considered to be necroptotic cells. Nec-1 pretreatment significantly reduced the number of TUNEL-positive cells but had no significant effect on cleaved caspase-3. Furthermore, Z-VAD pretreatment reduced few TUNEL-positive and cleaved caspase-3-positive cells. In the melatonin pretreatment group, TUNEL-positive cells were significantly reduced while cleaved caspase-3-positive cells were not decreased. Therefore, we infer that necroptosis was predominantly affected 6 h after CCI, which could be attenuated by Nec-1 and melatonin.

### Neuroprotective Effects of Nec-1, Z-VAD and Melatonin Following CCI

To verify the gross morphological effects of Nec-1, Z-VAD, and melatonin following CCI, we measured the cortical-lesion volumes of each group of rats. The volumes were significantly decreased by treatment with Nec-1 and melatonin (Supplementary Figures S4A,B). However, Z-VAD could not attenuate the lesion volume significantly, suggesting that necroptosis was involved predominantly in CCI early phase. Furthermore, a balance beam assay showed that Nec-1, melatonin, and Z-VAD all contributed to recovery of motor ability within 4 days after CCI, although the effect of Z-VAD was somewhat delayed, suggesting that caspase-dependent processes are not significantly activated in the early period after CCI and that reduction of early neural death is beneficial to the recovery of motor ability (Supplementary Figure S4C). Additionally, Nec-1 and melatonin, but not Z-VAD, decreased the edema in brain tissues at 6 h after CCI (Supplementary Figure S4D). To assess the effects of necroptotic inhibition on learning and memory ability, a MWM test was performed on days 15–20 after CCI, in which the time to reach a hidden platform was measured. Nec-1, melatonin, and Z-VAD treatments all improved the learning and memory deficits induced by CCI. Nec-1 and melatonin had significant effects as early as day 17, whereas the effect of Z-VAD was slightly delayed and did not show the same effects until day 18 (Supplementary Figure S4E). In order to eliminate the influences of differences in motivational deficits, swimming speed, and/or motor ability, the latencies for rats to reach a visible platform were tested. There was no significant difference among the groups in terms of the latency for rats to reach a visible platform. These findings are consistent with a neuroprotective role of Nec-1 and melatonin that is related, at least in part, to their ability to attenuate necroptosis in the early period after CCI.

### Suppression of Necroptosis After CCI Is Accompanied by Trafficking of HMGB1 From the Cytoplasm to the Nucleus

**Figure 4:**
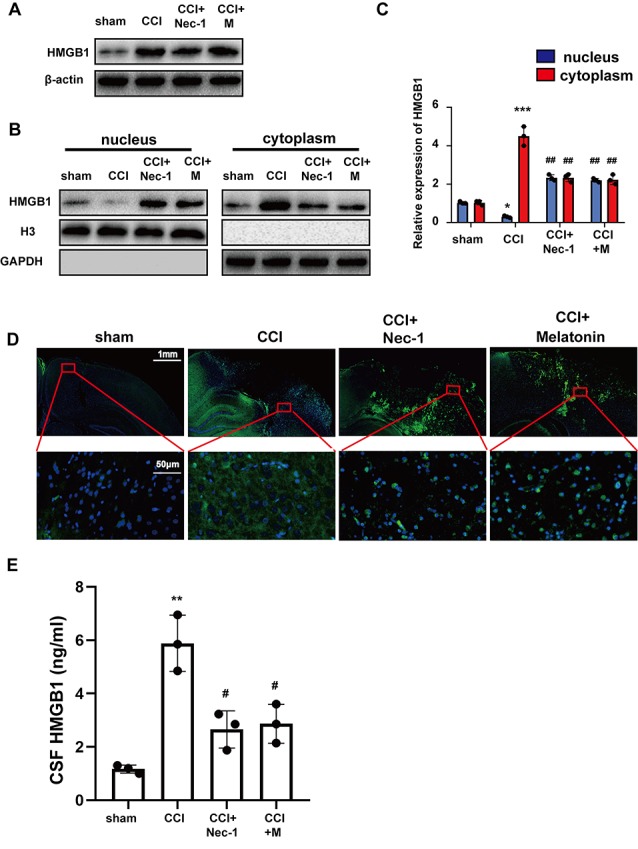
Nec-1 and melatonin attenuated inflammatory responses by blocking high-mobility group box-1 (HMGB1) translocation from the nucleus to the cytoplasm and release. **(A)** Total levels of HMGB1 after CCI tested by western blot were not decreased by Nec-1 or melatonin. **(B)** Tissues were fractionated, and the nuclear and cytoplasmic extracts were subjected to immunoblotting. GAPDH and H3 were used as controls for the cytoplasm and nucleus, respectively. **(C)** Relative expression in cytoplasm and nucleus was analyzed respectively by statistical. Values are represented as means ± SEM (*n* = 3). **(D)** The expression of HMGB1 was analyzed by immunofluorescence assays with the indicated antibodies. Samples were stained with an anti-HMGB1 antibody (green) and DAPI (blue). **(E)** Released HMGB1 in cerebrospinal fluid (CSF) was tested by Elisa assays. Relative level in CSF was analyzed by statistical. Values are represented as means ± SEM (*n* = 3). All experiments were analyzed by one way ANOVA plus Tukey’s test. *P < 0.05; **P < 0.01 and ***P < 0.001 vs. sham group. ^#^*P* < 0.05 and ^##^*P* < 0.01 vs. CCI group.

Previous studies have suggested that HMGB1 is trafficked from the nucleus to the cytoplasm and is then released extracellularly to bind to related receptors and induce inflammatory factors (Bi et al., 2017; Wen et al., 2017). To verify that Nec-1 and melatonin attenuate inflammation, we assessed the effect on HMGB1 after CCI. Western blotting demonstrated that CCI increased the expression of HMGB1, which was not significantly reduced by Nec-1 or melatonin (Figure 4A). Thus, we investigated the effects of CCI and an inhibitor of necroptosis on HMGB1 localization using Western blotting of nuclear and cytoplasmic extracts (Figures 4B,C). HMGB1 was expressed at similar levels in the nuclear and cytoplasmic extracts in the sham group but was predominately cytoplasmic in the CCI group. However, Nec-1 significantly attenuated the effect of CCI on the increased cytoplasmic HMGB1 localization, which was similar to the effect of melatonin. These results suggest that inhibition of necroptosis has a minimal effect on the total expression of HMGB1 but attenuates the increase in its cytoplasmic localization induced by CCI. These findings were confirmed by immunofluorescent assays, which revealed a predominant cytoplasmic localization of HMBG1 in the CCI group and a predominant nuclear localization of HMGB1 in the CCI+Nec-1 and CCI+M groups (Figure 4D). Elisa assays tested that abundant HMGB1 in CCI group released into CSF, which was alleviated by Nec-1 and melatonin (Figure 4E).

### RAGE and the Nuclear Factor (NF)-κB Pathway Are Involved in Necroptosis-Induced Neuroinflammation

**Figure 5:**
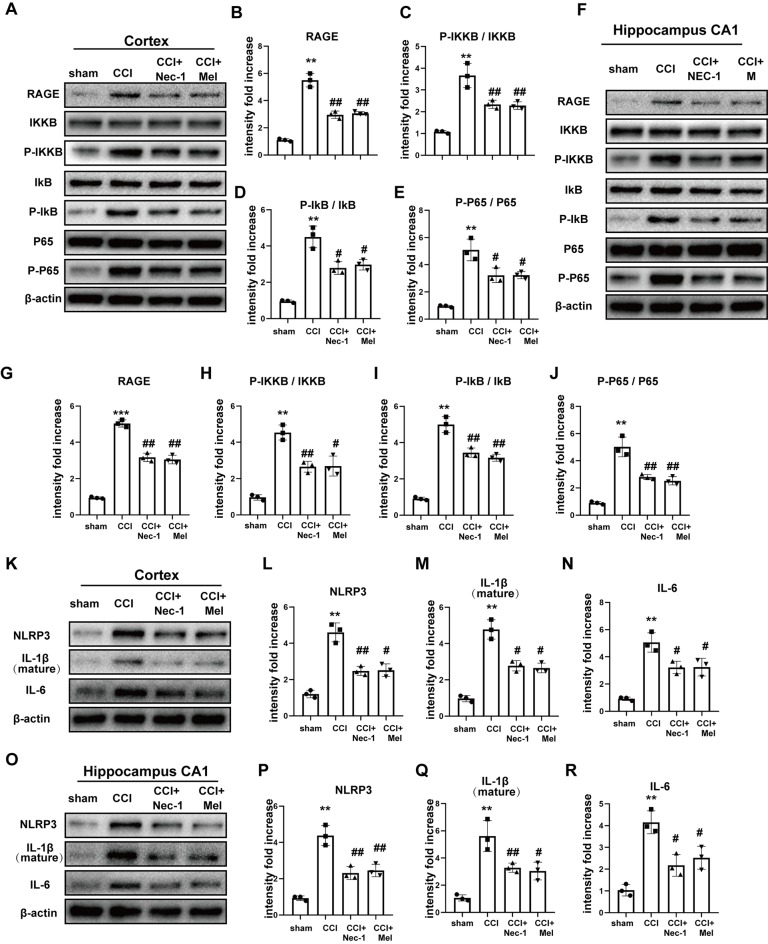
Downstream receptor for advanced glycation end-products (RAGE) and nuclear factor (NF)-κB pathway were inhibited after treatment of Nec-1 and melatonin. NF-κB pathway was detected by immunoblotting in the cortex **(A–E)** and hippocampus CA1 **(F–J)**. Relative downstream inflammatory factors were detected by immunoblotting in the cortex **(K–N)** and hippocampus CA1 **(O–R)**. All experiments were performed in triplicate by one way ANOVA plus Tukey’s test. **P < 0.01 and ***P < 0.001 vs. sham group. ^#^*P* < 0.05 and ^##^*P* < 0.01 vs. CCI group.

To further confirm neuroinflammatory levels following CCI, we assessed the expression of RAGE and the nuclear factor (NF)-κB pathway in the cortex and hippocampal CA1. RAGE levels were clearly increased after CCI, but these increases were inhibited by treatment with Nec-1 and melatonin. Furthermore, CCI promoted the phosphorylation of IKKB, IκB, and p65, and these CCI-induced increases were attenuated in the cortex (Figures 5A–E) and CA1 (Figures 5F–J) *via* Nec-1 and melatonin treatment. Inhibition of necroptosis by Nec-1 and melatonin also attenuated CCI-induced increases in NLRP3, IL-6, and IL-1β in the cortex (Figures 5K–N) and CA1 (Figures 5O–R), which demonstrated that these treatments inhibited the CCI-induced release of inflammatory cytokines.

### Silencing of A20 Exacerbates CCI-Induced Necroptosis

**Figure 6:**
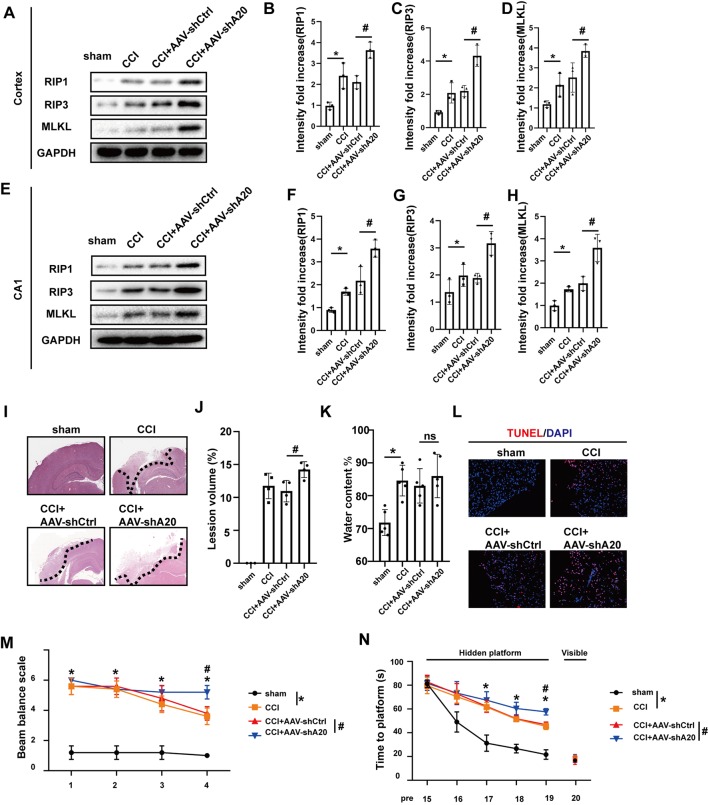
AAV-shA20 aggravated necroptosis and injury after CCI. **(A)** RIP1, RIP3 and MLKL in cortex were tested by western blot. **(B–D)** Data were analyzed by statistical. Values are represented as means ± SEM (*n* = 3). **(E)** RIP1, RIP3 and MLKL in hippocampus CA1 were tested by western blot. **(F–H)** Data were analyzed by statistical. Values are represented as means ± SEM (*n* = 3). **(I)** H&E showed the damaged area. **(J)** Lesion volume (*n* = 4) and **(K)** water content% (*n* = 5) were analyzed by statistical. Values are represented as means ± SEM. **(L)** TUNEL assays. **(M)** Ability of rats to remain on the balance beam among the four groups. Values are represented as means ± SEM (*n* = 7). **(N)** Morris water maze (MWM) was performed (*n* = 7). Latencies for rats to locate hidden or visible platforms on days 15–20 after CCI were shown. For the balance beam and MWM assays, data were analyzed by two-way ANOVA for between whole group comparisons. One-way ANOVA followed by Tukey *post hoc* test was used to analyzed the difference of groups at the same time point (* or # was labeled upon the relative time points). Other data, including western blot data, lesion volume and water content (%), were measured by one way ANOVA plus Tukey’s test. *P < 0.05 vs. CCI group. ^#^*P* < 0.05 vs. CCI+AAV-shCtrl group. *ns* means no significant difference between CCI+AAV-A20 and CCI+AAV-Ctrl group.

A20 (TNFAIP3) is thought to mediate cell death and the inflammatory response. A20 also adds lys48 chains to RIP1 *in vitro*, resulting in ubiquitination and degradation of RIP1, whereas non-ubiquitinated RIP1 promotes necroptosis when caspase activity is low (Vandenabeele et al., 2010; Vucic et al., 2011). However, little information is available on the role of A20 in TBI. In our CCI rat model of TBI, A20 was found to be upregulated following CCI, particularly at 6 h after injury (Supplementary Figures S5A–D). In order to explore whether A20 expression was involved in necroptosis, we silenced A20 *via* administration of AAV containing shRNA targeting A20 (AAV-shA20) and AAV-shCtrl. By using intracerebroventricular injections, the efficiencies of three AAV-shA20 sequences were measured in the cortex and CA1. The third sequence was the most efficient and, thus, was used in all subsequent experiments (Supplementary Figures S5E–H). RIP1, RIP3 and MLKL protein levels in the cortex (Figures 6A–D) and hippocampus CA1 (Figures 6E–H) were increased in the CCI+AAV-shA20 group compared to that in the CCI+AAV-ctrl group. Co-IP assays indicated that more RIP1 and MLKL could combine with RIP3 in CCI+ AAV-shA20 group (Supplementary Figures S7A–C, compare lane 1–2). It was found that more RIP1 and RIP3 combined with MLKL after AAV-shA20 administration (Supplementary Figures S7D–F, compare lane 1–2). In addition, more HMGB1 transferred to cytoplasm (Supplementary Figures S7G–J, compare lane 1–2) and then was released into extracellular CSF (Supplementary Figure S7K, compare lane 1–2). More damaged tissues and tissue-lesion volume were detected in the CCI+AAV-shA20 group (Figures 6I,J). However, AAV-shA20 administration did not exacerbate CCI-induced water content (%; Figure 6K). Finally, TUNEL assays indicated that A20 inhibition induced more cell death during CCI (Figure 6L). Rats in CCI+AAV-shA20 group performed a higher score than that in CCI+AAV-shCtrl, indicating A20 inhibition slowed the recovery of motor function (Figure 6M). WMW assays showed that rats in CCI+AAV-sh20 needed longer time to reach the invisible platform, indicating that silencing A20 aggravated the CCI-induced deficiency of learning and spatial memory ability (Figure 6N).

### A20 Downregulation Promotes Necroptosis *in vitro*

**Figure 7:**
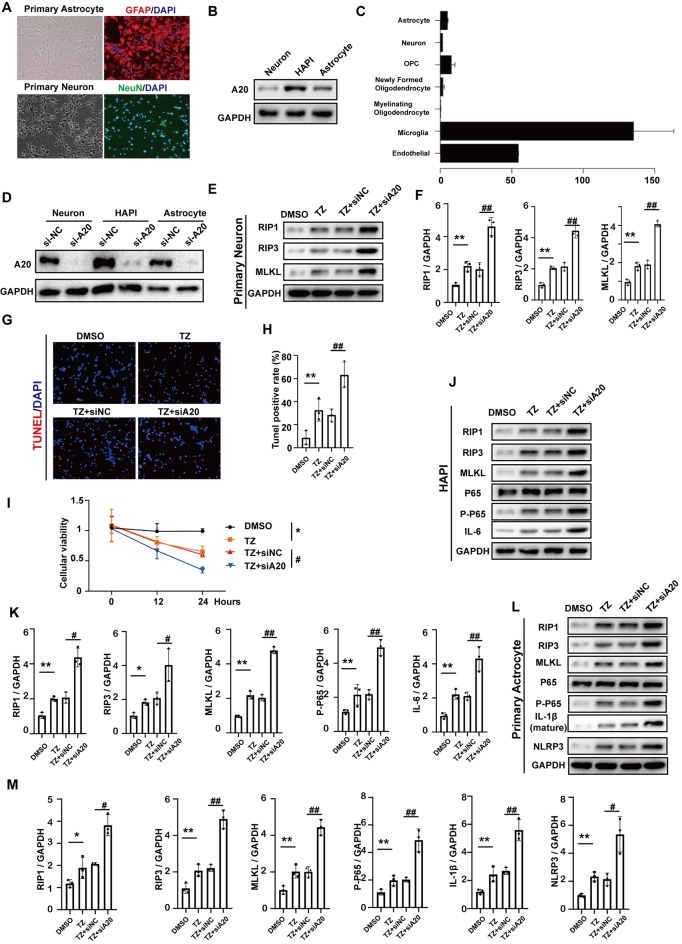
A20 downregulation promoted necroptosis *in vitro*. **(A)** Primary neuron and astrocyte were obtained and verified in relative antibody. **(B)** A20 protein expression in primary neuron, primary astrocyte and HAPI. **(C)** Relative mRNA expression of A20 in different cell types was obtained from a database of Stanford University (https://web.stanford.edu/group/barres_lab/brain_rnaseq.html). **(D)** A20 was significantly decreased after si-A20 treatment in the three cell types, including primary neuron, primary astrocyte and HAPI. **(E,F)** Tumor necrosis factor (TNF)-α (10 ng/ml) + Z-VAD (100 μm) combination (TZ) as a necroptosis inducer was used for 24 h. In primary neuron, RIP1, RIP3 and MLKL were tested by western blot. Data were analyzed by statistical. **(G,H)** TUNEL assays and **(I)** CCK-8 (*n* = 4) were used to test cell death and viability of neuron after 24 h treatment. **(J–M)** Western blot assay was used to test RIP1, RIP3, MLKL, NF-KB and relative inflammatory factors in primary HAPI and astrocyte respectively. Data were measured by one way ANOVA plus Tukey’s test. *P < 0.05 and **P < 0.01 vs. TZ group; ^#^*P* < 0.05 and ^##^*P* < 0.01 vs. TZ+si-NC group.

In order to investigate whether A20 influences RIP1, RIP3, MLKL and NF-KB expression in neurons, astrocytes and microglia, we used primary cortical neurons, primary astrocytes (Figure 7A) and HAPI, which is a rat microglial cell line *in vitro*. Western blotting assays indicated that the expression of A20 was highest in HAPI and lowest in neurons (Figure 7B), which is consistent with the results in the open-access database at Stanford University^1^ (Figure 7C). Next, TNF+Z-VAD (TZ) administration was used as a necroptotic inducer. After A20 small-interfering RNA (si-A20) cell lines, containing primary neurons, astrocyte and HAPI, were constructed (Figure 7D), RIP1, RIP3 and MLKL were tested. At the same time, si-NC was used as negative control. In primary neurons, RIP1, RIP3 and MLKL were increased after TZ. Si-A20 induced the much higher expression of RIP1, RIP3, and MLKL (Figures 7E,F). In the treatment of si-A20, a higher rate of TUNEL positive cells and lower cellular viability were detected (Figures 7G–I). In the HAPI, RIP1, RIP3, MLKL, p-p65, IL-6 were all increased under the condition of TZ, which were expressed much higher by si-A20 administration (Figures 7J,K). Similarly, TZ induced upregulation of RIP1, RIP3, MLKL, p-p65, IL-1β and NLRP3 were enhanced by silencing A20 (Figures 7L,M).

### A20 Is Required for Nec-1- and Melatonin-Mediated Reductions in RIP1 Levels

**Figure 8:**
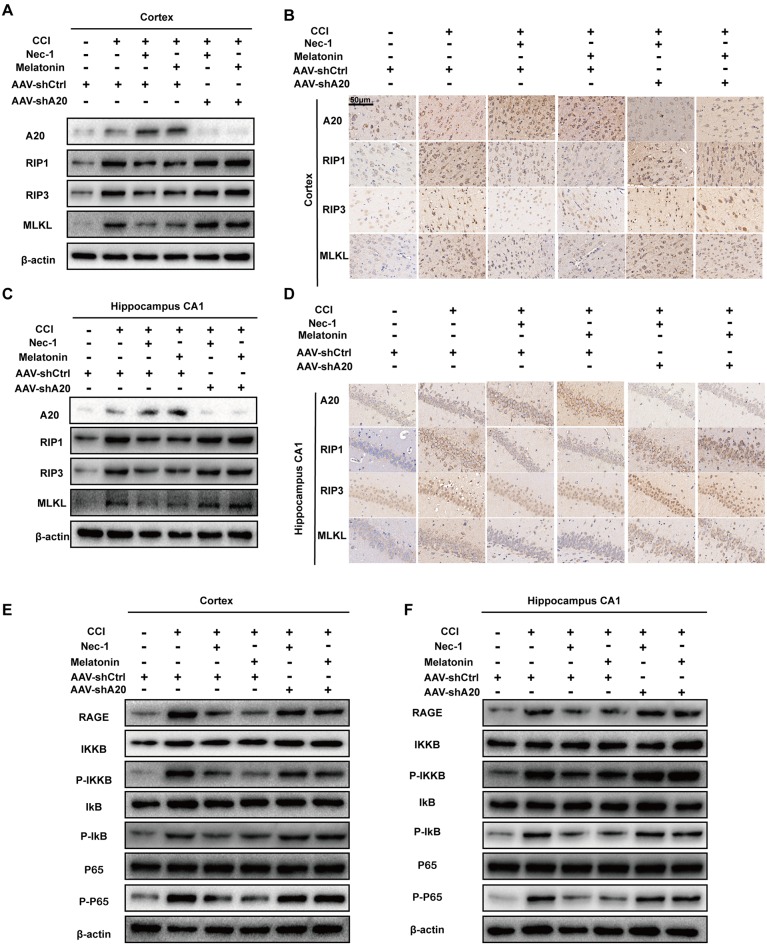
AAV-shA20 attenuated the anti-necroptotic roles of Nec-1 and melatonin and recovered the RAGE/NF-κB pathway. **(A,B)** In the cortex tissues, AAV-shA20 inhibited the effect of Nec-1 and melatonin on RIP1, RIP3 and MLKL, determined by immunoblotting and immunohistochemistry. β-actin was used as a control. **(C,D)** The same results were demonstrated in hippocampal CA1 tissues. **(E,F)** Restored RAGE and active NF-κB pathway were detected in cortex tissues by immunoblotting after AAV-shA20 administration. The same results were demonstrated in hippocampal CA1 tissues. β-actin was used as a control.

To determine the importance of A20 in alleviating CCI-induced necroptosis, we tested whether A20 levels alter the protective effects of Nec-1 and melatonin. qRT-PCR assays demonstrated that RIP1 mRNA levels were increased at 6-h post-CCI but were unaffected by AAV-shA20 (Supplementary Figures S5I,J). However, Western blotting demonstrated that AAV-shA20 counteracted the Nec-1- and melatonin-mediated reductions in protein expression of RIP1 in the CCI+Nec-1+AAV-shA20 and CCI+M+AAV-shA20 groups. In addition, two other components of the necrosome, RIP3 and MLKL, were both increased after lateral intracerebroventricular injection of AAV-shA20 into the cortex (Figure 8A and Supplementary Figures S6A–D) and CA1 (Figure 8C and Supplementary Figures S6E–H). This effect of AAV-shA20 on A20, RIP1, RIP3, and MLKL was verified by immunohistochemistry (Figures 8B,D). Co-IP assays demonstrated that more combination of RIP1/RIP3 and MLKL after A20 inhibition, indicating anti-necroptotic roles of Nec-1 and melatonin were alleviated (Supplementary Figures S7A–F, lane 3–lane 6). These results are consistent with a possible role of A20 in mediating inhibition of necroptosis. Additionally, western blot assays indicated silencing A20 could promote HMGB1 transfer into cytoplasm under the treatment condition of CCI+ Nec-1 or +melatonin (Supplementary Figures S7G–I, lane 3–lane 6). More HMGBI was also detected by immunofluorescence assays (Supplementary Figure S7J, lane 3 to lane 6). Increased HMGB1 in CSF was found in the AAV-shA20 administration group indicating that more HMGB1 was released out (Supplementary Figure S7K, lane 3–lane 6). RAGE and p-p65 were inhibited by Nec-1 and melatonin, but this decrease was attenuated by pre-treatment with AAV-shA20 in both the cortex (Figure 8E, Supplementary Figures S6I–L) and hippocampal CA1 (Figure 8F, Supplementary Figures S6M–P).

### AAV-shA20 Inhibits the Neuroprotective Role of Melatonin and Nec-1

We performed hematoxylin and eosin (H&E) assays and neurobehavioral experiments. While inhibition of necroptosis by Nec-1 or melatonin reduced lesions in brain tissues, A20 inhibition *via* AAV-shA20 injections reversed this effect (Supplementary Figures S8A,B). CCI-associated edema was reduced by Nec-1 or melatonin; again, this effect was inhibited by AAV-shA20 treatment (Supplementary Figure S8C). In inverted-screen tests, the time spent by rats suspended on the inverted screen was longer in the Nec-1+CCI+AAV-shCtrl and melatonin+CCI+AAV-Ctrl groups than that in the CCI+AAV-shCtrl group; however, it was shorter in the CCI+Nec-1+AAV-shA20 and CCI+melatonin+AAV-shA20 groups (Supplementary Figure S8D). In balance beam tests, the Nec-1- and melatonin-pretreated groups performed significantly better than the CCI+AAV-shCtrl group, but A20 inhibition impaired this ability (Supplementary Figures S8E,F). Furthermore, AAV-shA20 also significantly attenuated the beneficial effects of Nec-1 and melatonin in ameliorating cognitive and memory deficits at 15–20 days after CCI treatment, as assessed by latencies to reach the hidden platform in the MWM; importantly, the latencies to reach the visible platform were not significantly different among groups (Supplementary Figures S8G,H).

### AAV-shA20 Prevents the Attenuation of Inflammatory Responses After Treatment With Nec-1 or Melatonin in Rat CCI Model

**Figure 9:**
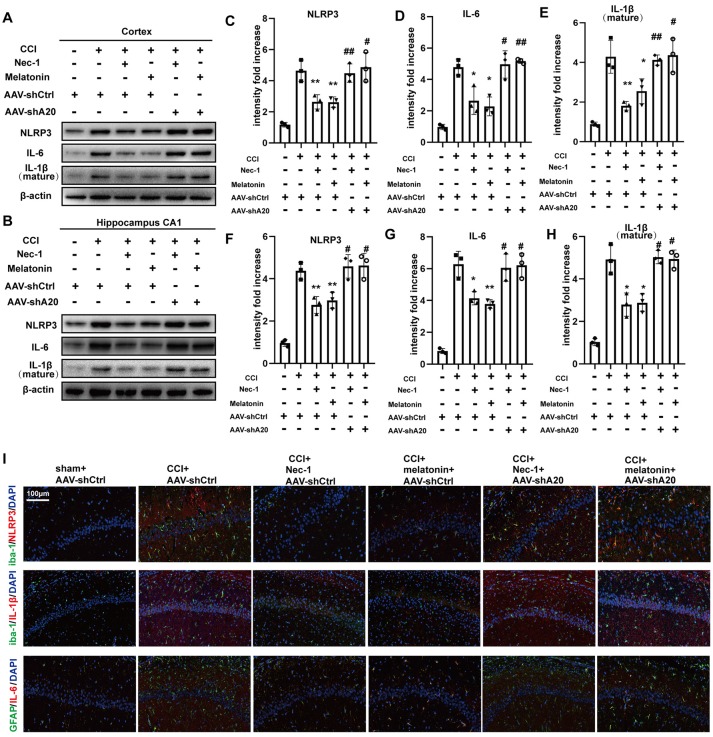
AAV-shA20 restored inflammatory responses. **(A,B)** Interleukin-6 (IL-6), interleukin-1 (IL-1β) and NLRP3 were detected in cortex tissues and hippocampal CA1 tissues by immunoblotting, respectively. The results including cortex **(C–E)** and hippocampus CA1 **(F–H)** were analyzed by statistical. **(I)** Representative images of double IL-1β (red) and Iba-1 (green) positive cells; NLRP3 (red) and Iba-1 (green); IL-6 (red) and GFAP (green) in the hippocampus CA1 location, as determined by immunofluorescence staining. Nuclei were stained with DAPI (blue). Data were measured by one way ANOVA plus Tukey’s test. *P < 0.05 and **P < 0.01 vs. CCI+AAV-shCtrl group. ^#^*P* < 0.05 and ^##^*P* < 0.01 vs. CCI+AAV-shCtrl+Nec-1 or CCI+AAV-shCtrl+melatonin group, respectively.

To determine whether A20 is also involved in inflammatory responses, we performed Western blotting of inflammatory proteins after AAV-shA20 treatment. NLRP3, IL-6, and IL-1β levels were reduced by Nec-1 and melatonin, but this reduction was reversed by AAV-shA20 in cortex (Figures 9A,C–E) and CA1 (Figures 9B,F–H). Thus, these results suggest that Nec-1- and melatonin-mediated inhibition of inflammatory responses is dependent on A20. Activated microglia and reactive astrocytes are key mediators used by the central nervous system to defend against injury and to activate immune and inflammatory responses (Guedes et al., 2014). We performed double-immunofluorescent staining using antibodies against the microglia marker, iba-1, and the astrocyte marker, GFAP, in combination with anti-NLRP3, -IL-1β, or -IL-6 (which is mostly produced by astrocytes). These markers were all increased by CCI, which suggests hyperplasia of microglia or astrocytes in the rat brain (Figure 9I). Mitigation of necroptosis by Nec-1 and melatonin attenuated these increases, and AAV-shA20 treatment reversed these effects. Taken together, these results indicate that attenuated necroptosis by Nec-1 and melatonin reduces CCI-induced levels of neuroinflammation, which was inhibited by silencing A20.

### A20 Downregulation Alleviates the Anti-necroptotic Effect of Nec-1 and Melatonin *in vitro*

In the primary neuron, Nec-1 and melatonin decreased the levels of RIP1, RIP3 and MLKL, but these decreases were inhibited in si-A20 cells (Supplementary Figures S9A, S10A–C). CCK-8 and TUNEL assays indicated that low expression of A20 promoted cell death and decreased cell viability (Supplementary Figures S9B,C, S10D). In HAPI cells (Supplementary Figures S9D, S10E–I) and primary astrocytes (Supplementary Figures S9E, S10J–O), RIP1, RIP3, MLKL, p-p65 and relative inflammatory factors were upregulated by TZ, which were alleviated by Nec-1 and Melatonin in an A20-dependent manner.

## Discussion

Necroptosis is a tightly regulated form of necrosis that can occur in a programmed manner under certain circumstances, such as during TBI-induced secondary injury. In the present study, we demonstrated that necroptosis occurred in human tissue after TBI. In our rat model of TBI, CCI-induced necroptosis and neuroinflammation were inhibited by Nec-1 and melatonin in an A20-dependent manner, and low A20 expression in CCI rats induced aggressive necroptosis and injury.

Necroptosis in TBI involves many cell types, such as neurons and glia. Necroptosis in neurons induces a programmed death process in response to injury; thus, motor ability and memory competence are impaired. Unlike neurons, glial cells secrete inflammatory factors when necroptosis occurs. Microglia and astrocytes are often mentioned in related studies (Gullo et al., 2017; Hu et al., 2018). Secreted inflammatory factors—such as TNF, IL-6, and IL-1—can recognize their cognate receptors on neuronal membranes and activate intercellular signaling pathways, further promoting neuronal death and attenuating motor ability and cognitive competence.

Previous studies have confirmed that TNF induces an important extracellular signaling pathway of necroptosis (Takahashi et al., 2012; Liao et al., 2017; Shen et al., 2017). After CCI, serum TNF is significantly increased and the cell-membrane expression of TNFR1 is upregulated (Yang et al., 2010). When TNF is recognized by TNFR1, TRADD and TRAF2 are recruited, which leads to the assembly of a proximal TNFR1-associated signaling complex containing c-IAP and RIP1. Although c-IAP1 ubiquitinates RIP1, the ubiquitination of RIP1 by c-IAP1 is not exclusive for necrosome formation (Vucic et al., 2011). A20 is an alternate molecule that modifies the polyubiquitylation of substrates *via* ubiquitin editing, which is the primary mechanism in apoptotic and inflammatory responses (Zheng et al., 2016). A previous study has shown that A20 attenuates TNF-induced NF-κB signaling by removing lys63 chains from RIP1 and adding degradative polyubiquitin lys48 chains *via* its zinc-finger 4 motif (Witt and Vucic, 2017). Non-ubiquitinated RIP1 associates with FADD and caspase-8 to form a secondary death-promoting signaling complex that dissociates from the receptor. When caspase activity is blocked or defective, non-ubiquitinated RIP1 binds RIP3 to promote necroptosis. In the early phase after CCI (6 h), we observed increased levels of RIP1 and other necrosome molecules but no significant change in cleaved caspase-3, suggesting that necroptosis may be significantly involved in the early phase after CCI. The pan-caspase inhibitor, Z-VAD, had little or no effect on necroptosis, which would be expected if necroptosis is the predominant form of cell death in the early period of CCI. Not like Z-VAD, melatonin inhibited necroptosis efficiently. A previous study showed that melatonin suppresses microglial necroptosis by regulating deubiquitinating (DUB) enzyme A20 after intracerebral hemorrhage (Lu et al., 2019). They also found that A20 inhibition could aggravate RIPK3-dependent necroptosis induced by ICH. However, caspase-dependent apoptosis was also inhibited by melatonin. In order to eliminate the effect of melatonin on anti-apoptosis, we tested necroptosis and inflammation at 6 h after CCI. It is worth noting that caspase-3 was not significantly increased in the early phase of CCI, although it increased dramatically after 24 h, suggesting that apoptosis might be initiated after necroptosis. Some studies indicated that preventing necroptosis after subsequent activation of apoptosis (12 or 24 h after CCI) by relative inhibitors could reduce cell death and improve recovery of brain lesions (Liu et al., 2018; Ni et al., 2019). In our view, necroptosis inhibition at those time points could influence other death mechanisms and pathways, such as apoptosis. Abundant studies have indicated possible and intricate relationship between necroptosis and apoptosis (Heckmann et al., 2019; Ruhl et al., 2019; Zhang et al., 2019). A study also demonstrated that repression of either of RIP3 or MLKL did not protect the cells from death, but instead induced a switch from TNF-induced necroptosis to RIP1 kinase-dependent apoptosis (Remijsen et al., 2014). Thus, the relationship between necroptosis and apoptosis might be complex and intricate.

Nec-1 and melatonin could inhibit HMGB1 translocation not only in CCI but in other organs’ diseases. In intestinal ischaemia/reperfusion injury, Nec-1 alleviated cytoplastic expression of HMGB1 but not total level, which reducing enterocytes loss (Wen et al., 2017). It was also found that melatonin promoted renal regeneration in acute kidney injury (Zhu et al., 2017) and alleviated circadian rhythm disruption by inhibiting the distribution of HMGB1 (Liu and Wang, 2019). Thus, HMGB1 is a significant mediator in necroptosis and inflammation. Inflammatory response after CCI was considered as a negative factor to neuronal repair. And relative studies have indicated that necroptosis contributed to inflammation, which promoted cell death (Pasparakis and Vandenabeele, 2015; Huang et al., 2018; Polykratis et al., 2019; Yang et al., 2019). However, Inflammatory response and necroptosis occurred in CCI in parallel. In CCI rat model, inflammatory response was mainly found in microglia and a small part in astrocyte, which could not be regulated by necroptosis completely. Thus, further studies should be performed to explore the inflammatory response in CCI.

The molecular mechanisms underlying necroptosis during TBI have remained largely unexplored. A20 (TNFAIP3) is an endogenous anti-inflammatory factor that is induced under various circumstances. A20 ameliorates intracerebral hemorrhage-induced inflammatory injury and intracerebral hemorrhage increases A20 expression, showing a peak after approximately one day (Takahashi et al., 2012); although these previous A20 results are similar to those of our present study, we found the A20 level was rapidly increased in our CCI model, which indicates differential A20 expression in different disease models. In the present study, AAV-shA20 did not influence the mRNA level of RIP1 but did increase its protein level, which indicates that A20 may be involved in RIP1 protein stability in our CCI model. A previous study also indicated that A20 functions as a ubiquitin-editing enzyme with both DUB and ubiquitin E3 ligase activity toward RIP1 in the TNFR pathway. A20 first cleaves lysine 63 (K63)–linked polyubiquitin chains on RIP1 and then conjugates lysine 48 (K48)–linked polyubiquitin chains that target RIP1 for degradation by the proteasome (Shembade et al., 2010). However, some studies indicated another mechanism mediated by A20 that A20s DUB motif is required for inhibiting RIPK3 ubiquitination and RIPK1-RIPK3 complex formation (Onizawa et al., 2015). Their studies link A20 and RIPK3 ubiquitination to necroptotic cell death, and suggest new mechanisms by which A20 may prevent inflammatory disease.

Our present study has several limitations. First, our results suggest that necroptosis is the main mechanism of cell death at 6 h after CCI, but the detrimental effects of CCI are much longer lived. We suspect that necroptosis may play a primary role in the early phase after CCI but that other cell death pathways—such as autophagy, pyroptosis, and apoptosis—mediate subsequent pathological processes. Thus, further study is needed to determine the relationship between these pathways and necroptosis after CCI.

**Figure 10:**
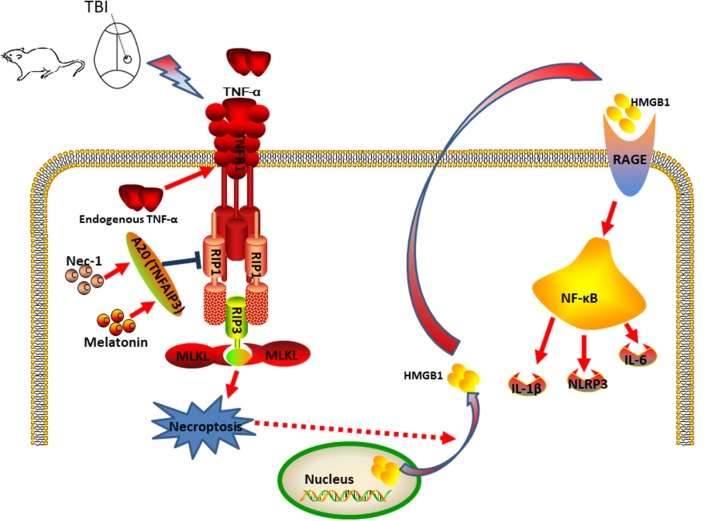
Putative mechanism of necroptosis and relative inflammatory responses during the development of CCI. In the CCI model, CCI stimulates TNF-α production and TNFR1 signaling, and activation of the downstream RIP1/RIP3-MLKL signaling pathway, which triggers necroptotic death and release of HMGB1 from the nucleus to the cytoplasm, further contributing to additional inflammatory factors. Nec-1 and Melatonin administration decreased necroptosis and extracellular HMGB1 levels, which decreased inflammation induced by RAGE expression and the detrimental effect of HMGB1 signaling in CCI. Thus, Nec-1 and melatonin inhibit the inflammatory responses and downstream NF-κB, NLRP3, IL-6 and IL-1β, contributing to neuroprotection. A20 also plays a role in this process and deficient expression of A20 could aggravate CCI-induced necroptosis. In addition, silencing A20 could impair the anti-necroptotic effect of Nec-1 and melatonin.

In summary, we propose a model in which CCI stimulates the production of TNF, which binds to TNFR1 and activates and recruits a protein complex composed of RIP1, RIP3, and MLKL to trigger necroptosis (Figure 10). We further propose that Nec-1 and melatonin reduce necroptosis and subsequently inhibit HMGB1, RAGE, and proinflammatory cytokines in an A20-dependent manner. Taken together, the findings of our present study suggest novel therapeutic targets for nervous system recovery after TBI.

## Data Availability Statement

Publicly available datasets were analyzed in this study. This data can be found here: https://web.stanford.edu/group/barres_lab/brain_rnaseq.html.

## Ethics Statement

The studies involving human participants were reviewed and approved by The Research Ethics Committee of Nanjing Medical University. The patients/participants provided their written informed consent to participate in this study. The animal study was reviewed and approved by Animal Ethical and Welfare Committee of NJMU, and protocol has been reviewed and approved by the Animal Ethical and Welfare Committee, and protocol has been reviewed and approved by the Animal Ethical and Welfare Committee (AEWC).

## Author Contributions

JJ designed this research. ZB and LF performed the experiment. XX and LZ provide the resource of human brain samples. YiL and HC provided the technical support. NL, YY, YaL and XW offered data analysis.

## Conflict of Interest

The authors declare that the research was conducted in the absence of any commercial or financial relationships that could be construed as a potential conflict of interest.

## Footnotes

^1^https://web.stanford.edu/group/barres_lab/brain_rnaseq.html

## Abbreviations

TBI: Traumatic brain injury
HMGB1: High-mobility group box-1
CCI: Controlled cortical impact
NBT: Normal brain tissues
IL-6: Interleukin-6
IL-1β: Interleukin-1
TUNEL: TdT-mediated dUTP Nick-End Labeling
AAV: Adeno-associated virus
MWM: Morris water maze
BBB: Blood-brain barrier.
