# Supplementary material for: Silencing of A20 Aggravates Neuronal Death and Inflammation After Traumatic Brain Injury: A Potential Trigger of Necroptosis
Source: Front Mol Neurosci. 2019 Sep 19;12:222. doi: 10.3389/fnmol.2019.00222 (PMC6761256; doi:10.3389/fnmol.2019.00222)
Supplement: Supplementary file 1 [file Table_1.docx]

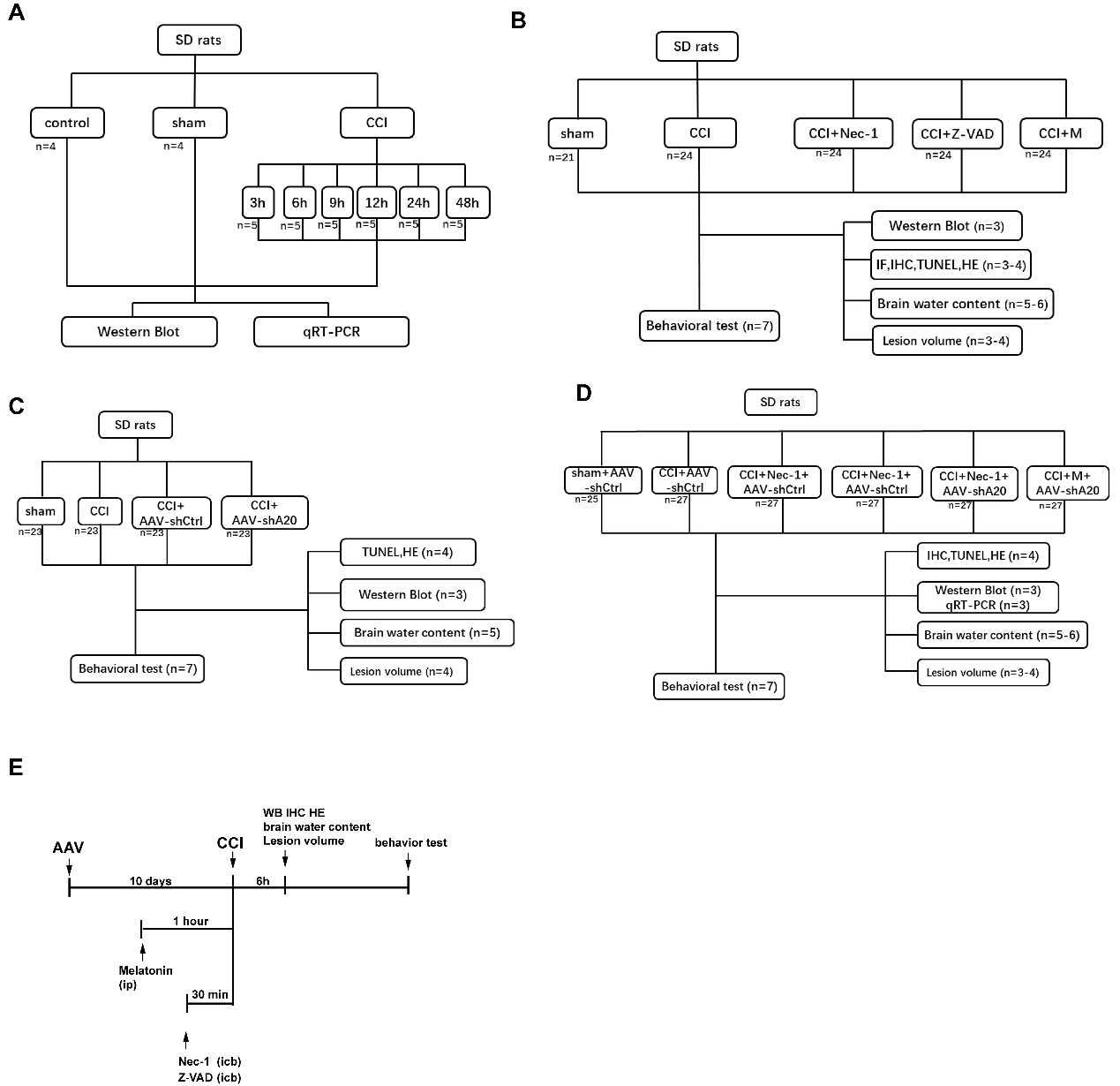
**Supplementary Figure 1**

Supplementary Figure 1.

Experimental design. (A) Experiment 1 was designed to detect the time course of necroptosis after CCI. (B) Experiment 2 was designed to detect the effects of necroptosis inhibition on CCI induced injury and investigate the possible mechanism. (C) Experiment 3 was designed to detect the effect of A20 on CCI induced necroptosis. (D) Experiment 4 was designed to detect the effect of A20 on anti-necroptotic effect of Nec-1 and melatonin. (E) A schedule for AAV administration and drug treatment for experiment design 4

**
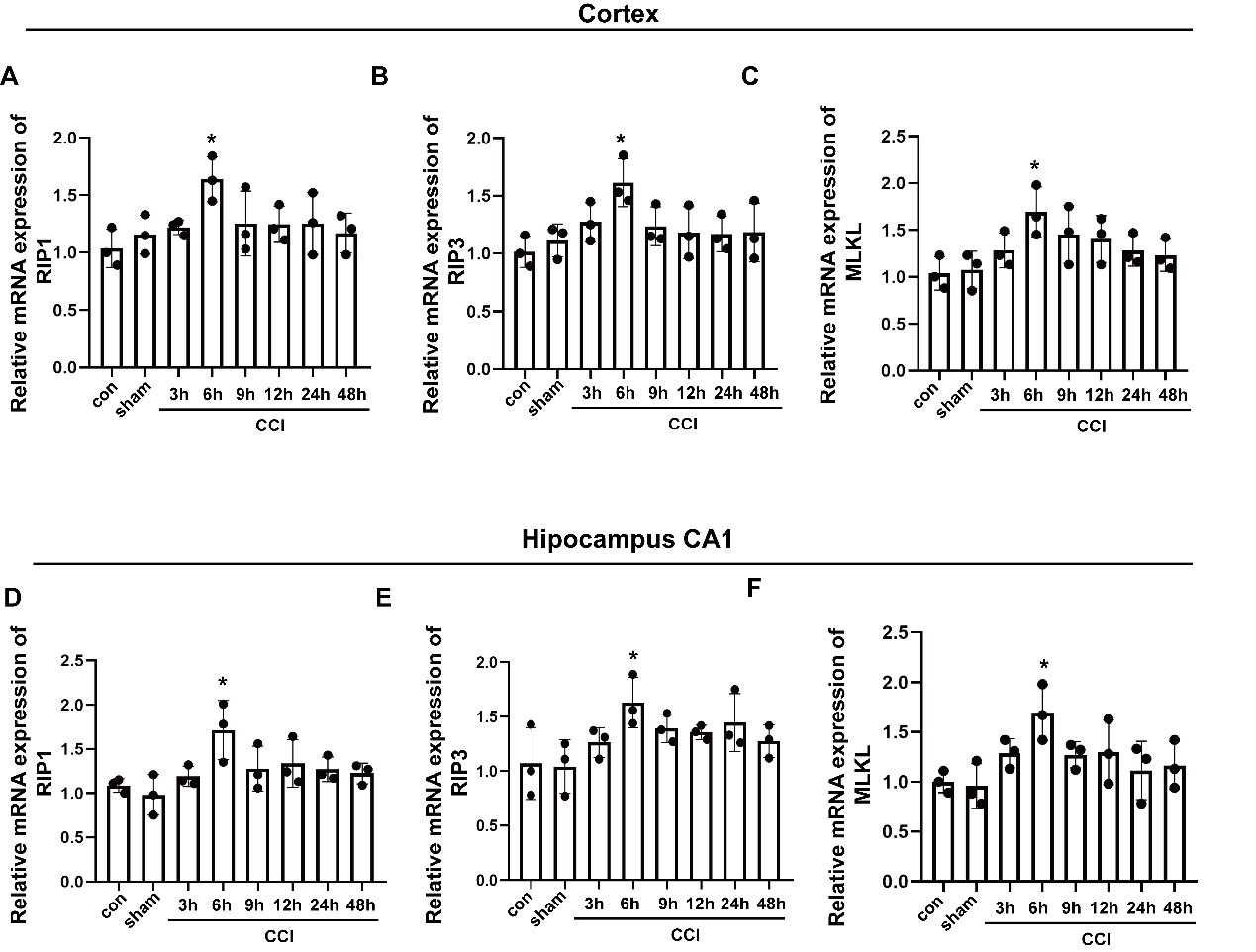
Supplementary Figure 2**

Supplementary Figure 2.

RNA levels of RIP1, RIP3 and MLKL after CCI. They were tested by qPCR assays in cortex (A-C) and hippocampus CA1(D-F). Values are represented as means ± SEM. (n=3). All experiments were analyzed by one way ANOVA plus Tukey's test. * P<0.05 versus sham group.

**Supplementary Figure 3**


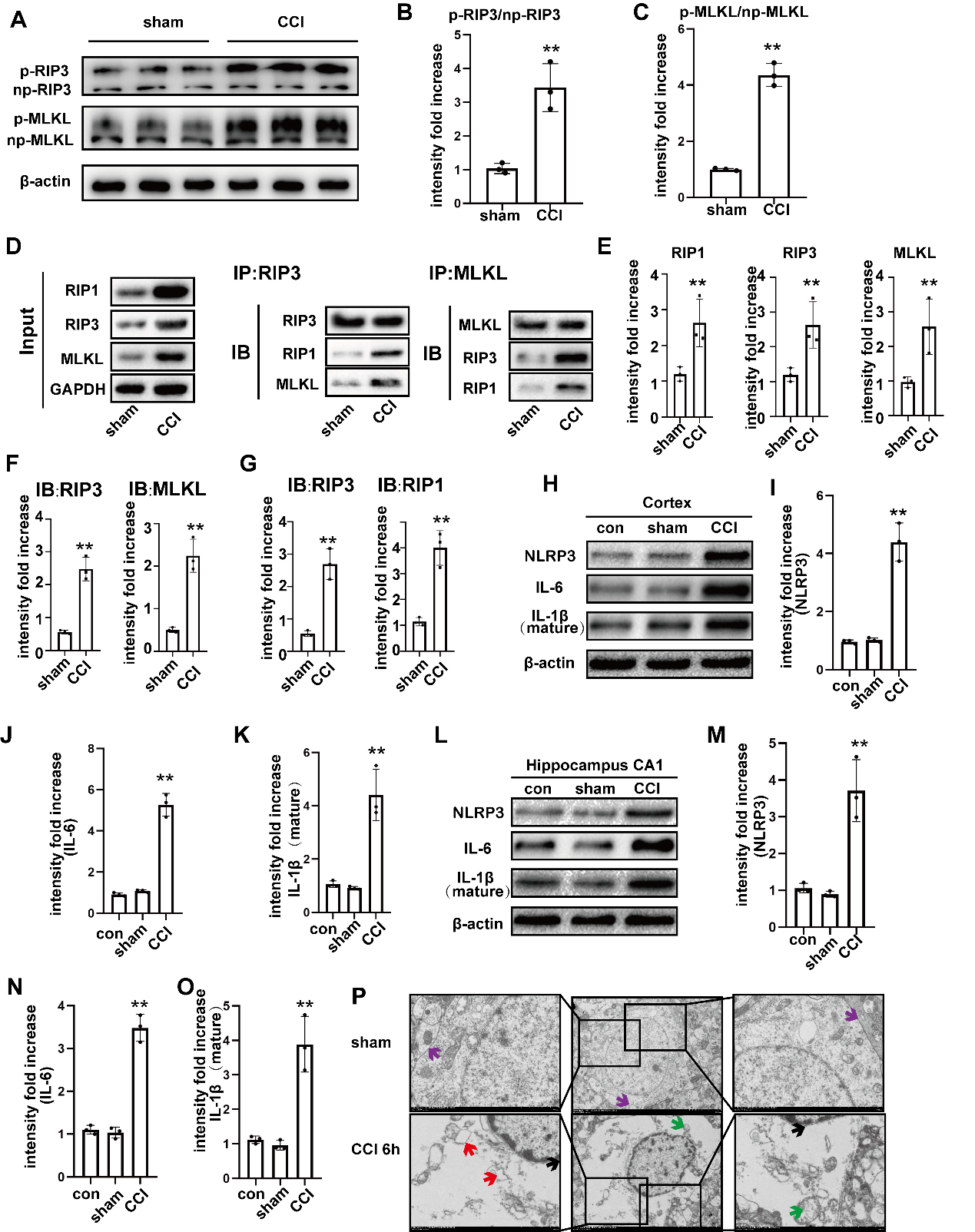


Supplementary Figure 3. Inflammatory responses were combined with necroptosis caused by CCI in rats. (A-C) Phosphorylation of RIP3 and MLKL are increased after CCI. Phos-tag™ SDS–PAGE was used in this experiment, in which phosphorylation of RIP3 and MLKL could be detective. β-actin was used as a control. (D) Co-immunoprecipitation assays were used to demonstrate the combination of RIP1/RIP3 and MLKL at 6 h after CCI. (E-G) Western blot data were analyzed by statistical. Values are represented as means ± SEM. (n=3). Inflammatory factors including NLRP3, IL-6 and IL-1β were detected in control, sham and at 6 h after CCI groups, by western blotting of cortex(H-K) and hippocampus CA1(M-O) tissues. Western blot data were analyzed by statistical. Values are represented as means ± SEM. (n=3). (P) Electron microscopy was used to examine normal brain tissues and brain tissues at 6 h after CCI. Intact cell membrane (violet) is labeled in NBT. Complete and continuous nuclear membrane (black arrow), swollen mitochondria (green arrow) and vacuoles (red arrow) were labeled in TBI tissues. All experiments were analyzed by one way ANOVA plus Tukey's test. ** P < 0.01 versus sham group.

**
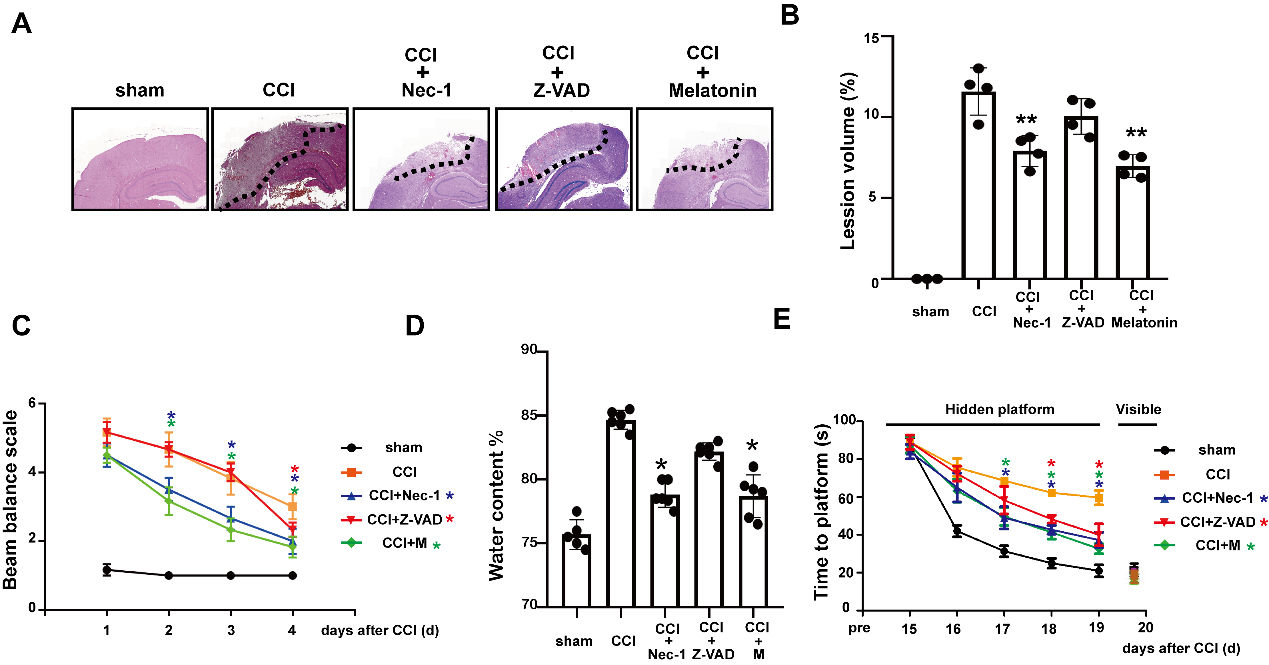
Supplementary Figure 4**

Supplementary Figure 4. Effect of Nec-1, Z-VAD and melatonin on the neuroprotective responses. (A) HE assays analyzed the lesion volume. (B) Statistical results of lesion volume showed a neuroprotective effect of Nec-1, and melatonin. Values are represented as means ± SEM. (n=3-4). (C) Ability of rats to remain on the balance beam among the 5 groups. Values are represented as means ± SEM. (n=7). (D) Brain water content after CCI was tested to evaluate the degree of inflammatory edema. Values are represented as means ± SEM. (n=5-6). Data, including lesion volume and water content (%), were measured by one way ANOVA plus Tukey's test. * P<0.05 and ** P < 0.01 versus CCI group. (E) Morris water maze (MWM) was performed (n=7). Latencies for rats to locate hidden or visible platforms on days 15-20 after CCI were shown. For the balance beam and MWM assays, data were analyzed by two-way ANOVA for between whole group comparisons. One-way ANOVA followed by Tukey post hoc test was used to analyzed the difference of groups at the same time point (* was labeled upon the relative time points). Among of them, *(green) means CCI+melatonin group vs CCI group; *(red) means CCI+Z-VAD group vs CCI group and *(blue) means CCI+Nec-1 vs CCI group.


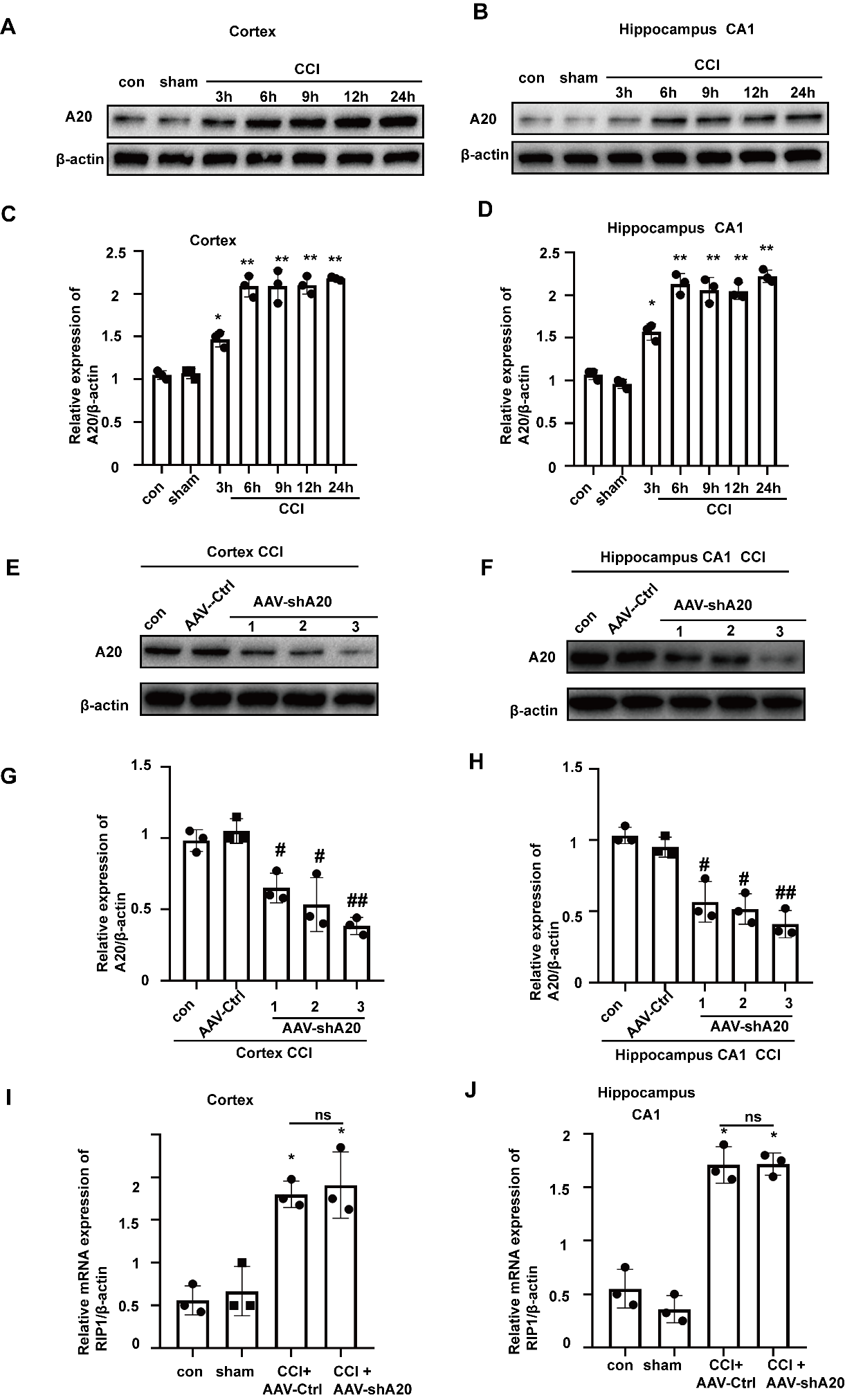
**Supplementary Figure 5**

Supplementary Figure 5. Changes of A20 have little effect on mRNA expression of RIP1. (A-B) In the cortex and hippocampus CA1, expression of A20 was increased after CCI treatment. (C-D) The result was analyzed by statistical. β-actin was used as a control. Values are represented as means ± SEM. (n=3). (E-F) In the cortex and hippocampus CA1, CCI induced the expression of A20. Efficiency of AAV-shA20 was verified and the third one was the most efficient. (G-H) The result was analyzed by statistical. Values are represented as means ± SEM. (n=3). (I-J) In the cortex and hippocampus CA1, CCI increased mRNA expression of RIP1. AAV-shA20 had little effect on it. The result was analyzed by statistical. * P<0.05 and ** P < 0.01 versus sham group. #P<0.05 and ##P < 0.01 versus CCI+AAV-Ctrl group. *ns* means no significant difference between CCI+AAV-A20 and CCI+AAV-Ctrl group. All experiments were analyzed by one way ANOVA plus Tukey's test.

**Supplementary Figure 6**


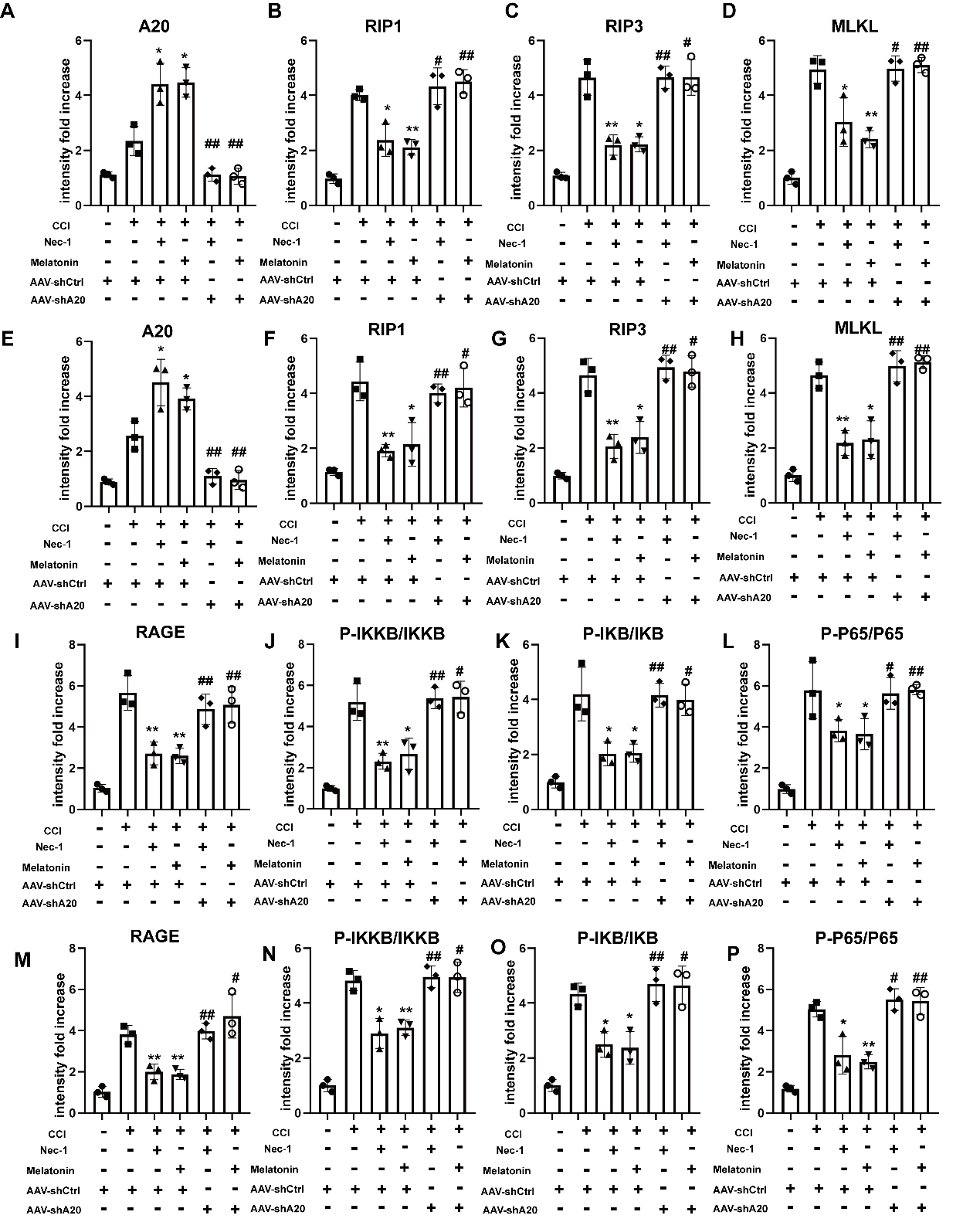


Supplementary Figure 6. Statistical Analysis of western blot result in Figure 8. (A-D) The statistic results of Figure 8 A. (E-H) The statistic results of Figure 8 C. (I-L) The statistic results of Figure 8 E. (M-P) The statistic results of Figure 8 F. The result was analyzed by statistical. Values are represented as means ± SEM. (n=3). * P<0.05 and ** P < 0.01 versus CCI+AAV-shCtrl group. #P<0.05 and ##P < 0.01 versus CCI+AAV-shCtrl+Nec-1 or +Melatonin group respectively. All experiments were analyzed by one way ANOVA plus Tukey's test.

Supplementary Figure 7


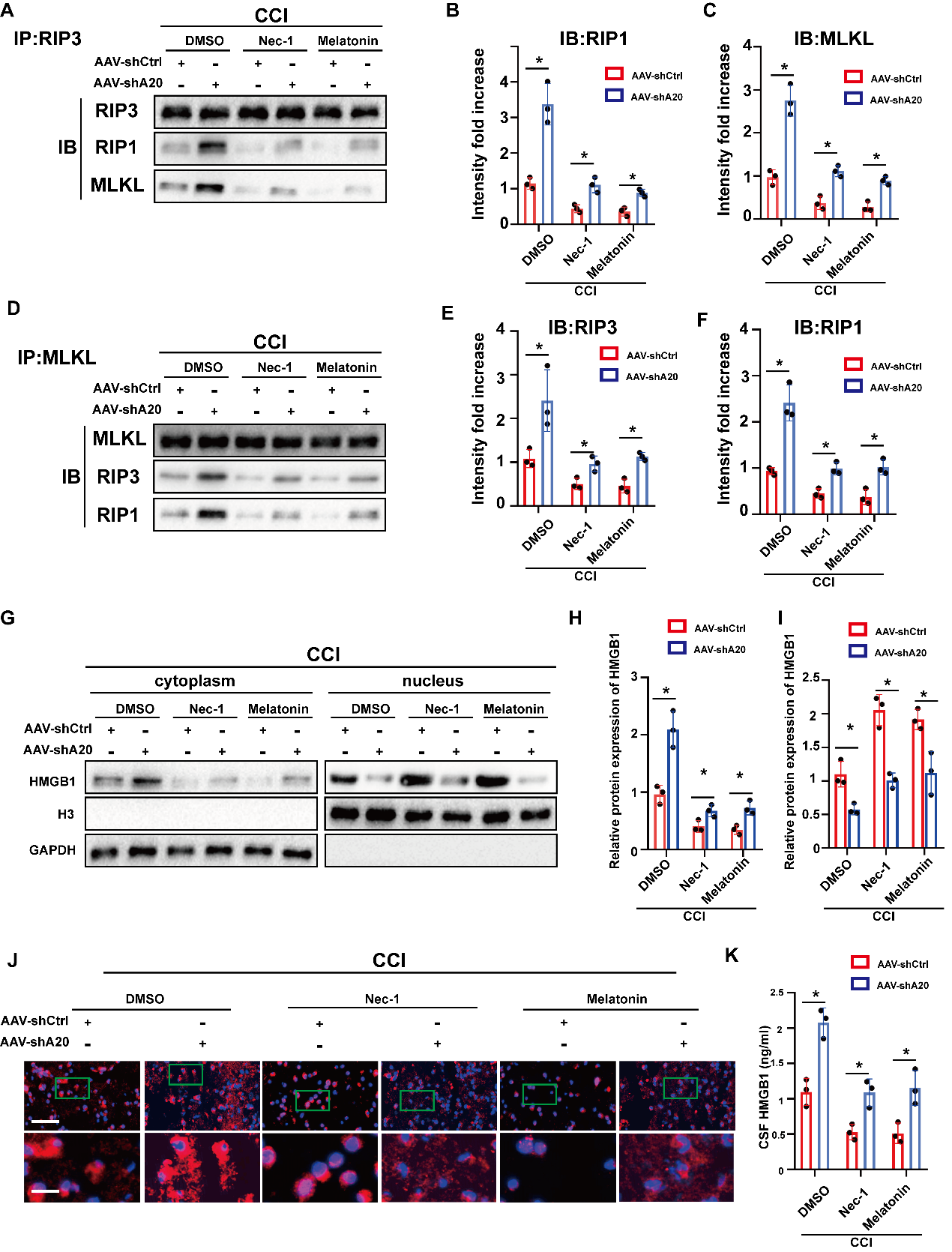


Supplementary Figure 7. AAV-shA20 administration promoted combination of RIP1/RIP3 and MLKL and transferring of HMGB1 from nucleus to cytoplasm after CCI. (A) Co-IP assays showed the combination of RIP1 and MLKL with RIP3. (B-C) The results were analyzed by statistical. Values are represented as means ± SEM. (n=3). (D) Co-IP assays showed the combination of RIP1 and RIP3 with MLKL. (E-F) The results were analyzed by statistical. Values are represented as means ± SEM. (n=3). (G-I) Under the three conditions of CCI+DMSO, CCI+Nec-1 and CCI+melatonin groups, western blot assays indicated that AAV-shA20 promoted HMGB1 to transfer into cytoplasm. Western blot data were analyzed by statistical. Values are represented as means ± SEM. (n=3) (J) Location of HMGB1(red) was detected by IF. Nucleus was stained by DAPI (Blue) (K) Elisa assays was used to test released HMGB1 in CSF. The result was analyzed by statistical. Values are represented as means ± SEM. (n=3). * P<0.05 versus AAV-shCtrl group under the three conditions of CCI+DMSO, CCI+Nec-1 and CCI+melatonin.

**Supplementary Figure 8**


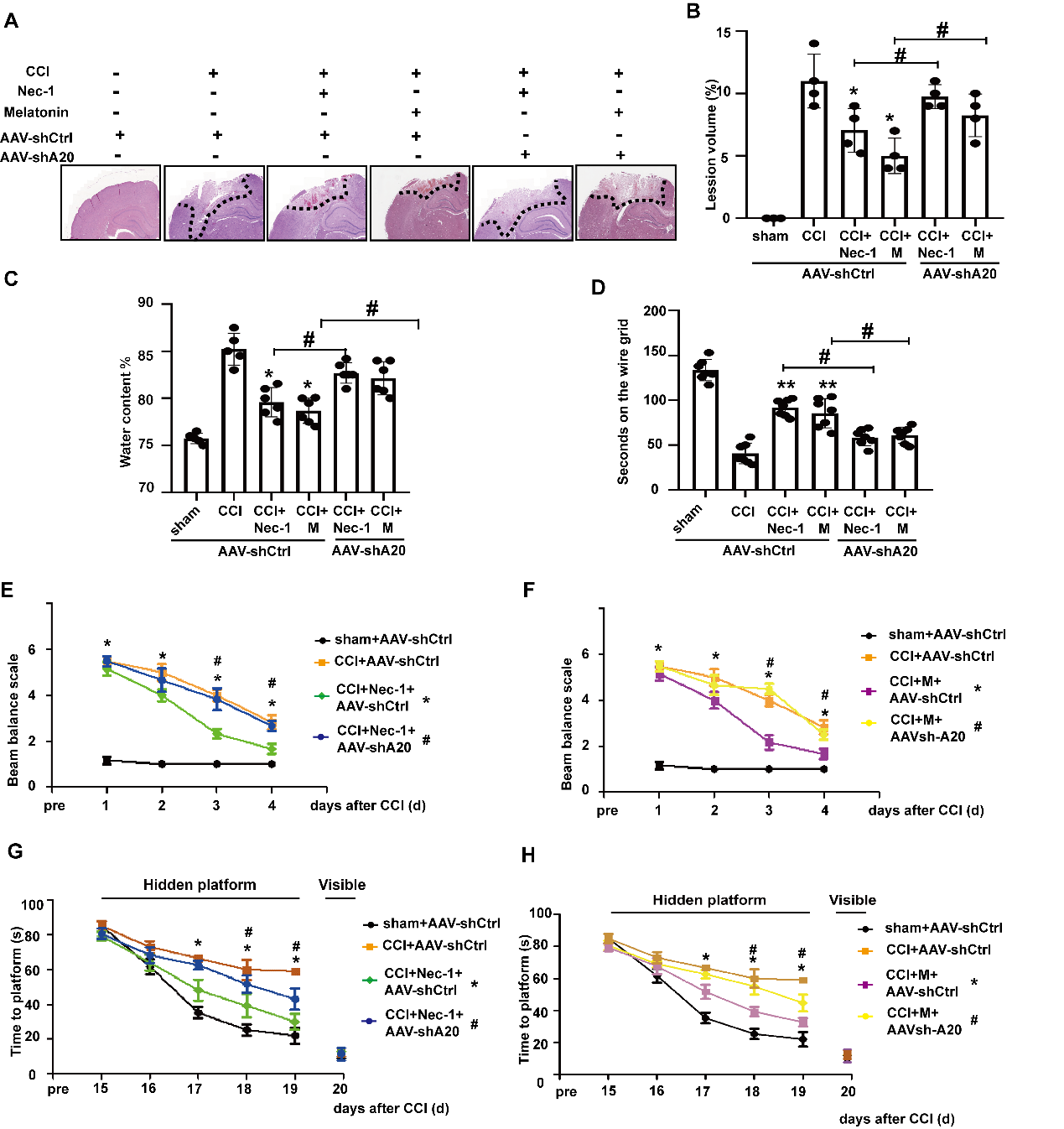


Supplementary Figure 8. AAV-shA20 inhibited the neuroprotective role of melatonin and Nec-1. (A) HE assays showed augmented damaged area after AAV-shA20 treatment. (B) Statistical analysis of lesion volume(%) results. Values are represented as means ± SEM. (n=4). (C) Brain water content after treatment was tested to evaluate the degree of inflammatory edema. Values are represented as means ± SEM. (n=5-6). (D) Time on the wire grid after AAV-shA20 treatment was tested. Values are represented as means ± SEM. (n=7). (E-H) Ability of rats to remain on the balance beam was tested. Values are represented as means ± SEM. (n=7). (G-H) Morris water maze (MWM) was performed. Latencies for rats to locate hidden or visible platforms on days 15-20 after CCI were shown. Values are represented as means ± SEM. (n=7). For the balance beam and MWM assays, data were analyzed by two-way ANOVA for between group comparisons. One-way ANOVA followed by Tukey post hoc test was used to analyzed the difference of groups at the same time point (* or # was labeled upon the relative time points). Other data, including lesion volume(%), water content(%) and seconds on the wire grid were measured by one way ANOVA plus Tukey's test. * P<0.05 and ** P < 0.01 versus CCI+AAV-shCtrl group; #P<0.05 versus CCI +AAV-shCtrl+ Nec-1 or +melatonin group respectively.

**Supplementary Figure 9**


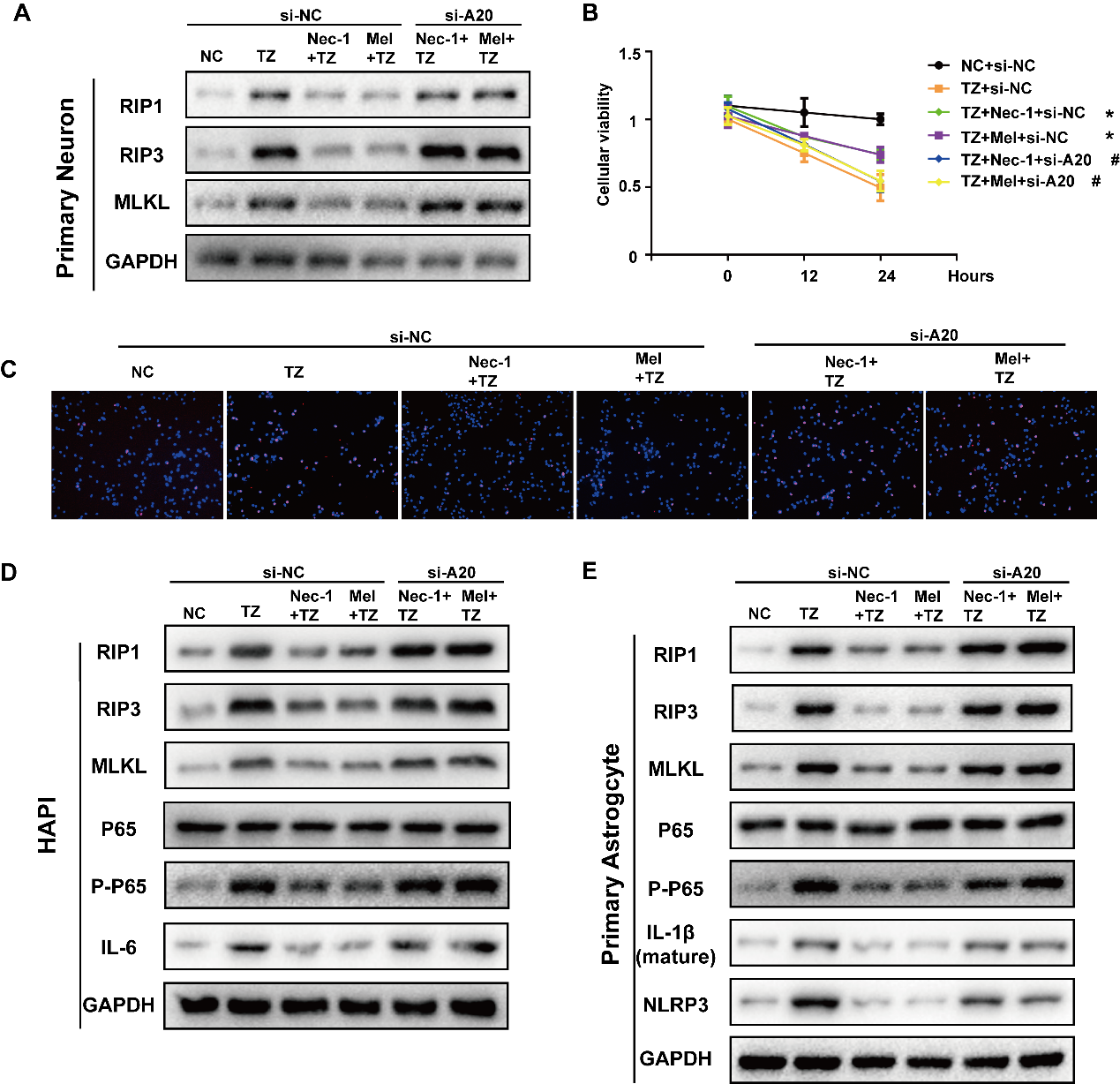


Supplementary Figure 9. A20 downregulation alleviated anti-necroptotic effect of Nec-1 and melatonin in vitro. (A) TNF-α (10 ng/ml)+Z-VAD (100 um) combination (TZ) as a necroptosis inducer was used for 24 hours. In primary neuron, RIP1, RIP3 and MLKL were tested by western blot. (B) CCK-8 (n=4) and (C) TUNEL assays were used to test cell viability and death of neuron after 24 hours treatment. (D, E) Western blot assay was used to test RIP1, RIP3, MLKL, NF-KB and relative inflammatory factors in primary HAPI and astrocyte. Data were measured by one way ANOVA plus Tukey's test. * P<0.05 versus TZ+ si-NC group; #P<0.05 versus TZ +si-NC +Nec-1 or +melatonin groups respectively.

**Supplementary Figure 10**


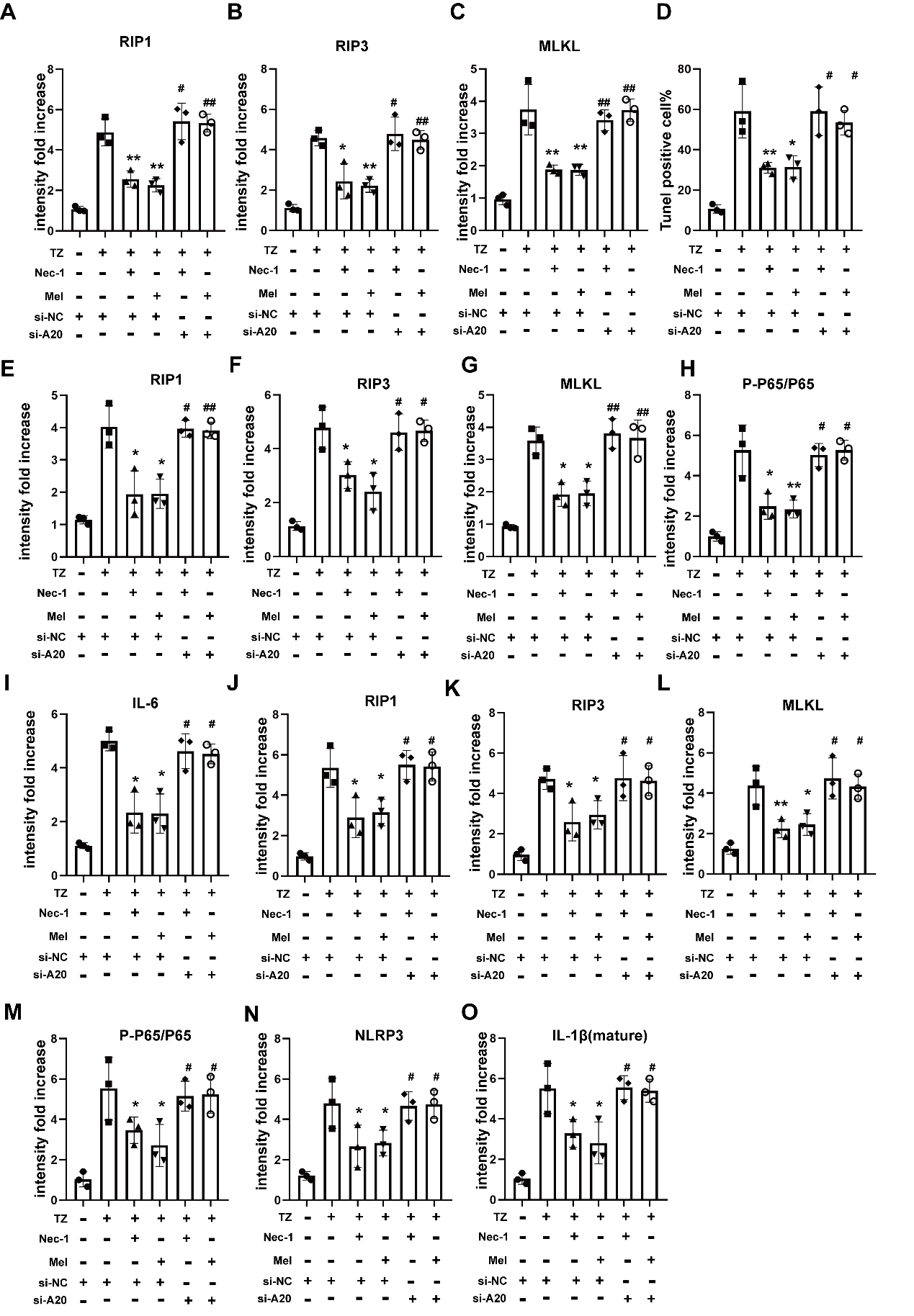


Supplementary Figure 10. Statistical Analysis of relative results in Supplementary Figure 9. (A-C) The statistic results of Supplementary Figure 9A. (D) TUNEL-positive neuron was analyzed. (E-I) The statistic results of Supplementary Figure 9D. (J-O) The statistic results of Supplementary Figure 10E. Data were measured by one way ANOVA plus Tukey's test. Values are represented as means ± SEM. (n=3). *P<0.05 and **P < 0.01 versus TZ +si-NC group; #P<0.05 and ##P < 0.01 versus TZ +si-NC+ Nec-1 or +melatonin group respectively.
